# Assessing the energy trap of industrial agriculture in North America and Europe: 82 balances from 1830 to 2012

**DOI:** 10.1007/s13593-023-00925-5

**Published:** 2023-11-08

**Authors:** Enric Tello, Vera Sacristán, José R. Olarieta, Claudio Cattaneo, Joan Marull, Manel Pons, Simone Gingrich, Fridolin Krausmann, Elena Galán, Inés Marco, Roc Padró, Gloria I. Guzmán, Manuel González de Molina, Geoff Cunfer, Andrew Watson, Joshua MacFadyen, Eva Fraňková, Eduardo Aguilera, Juan Infante-Amate, Alexander Urrego-Mesa, David Soto, Lluis Parcerisas, Jérôme Dupras, Lucía Díez-Sanjuán, Jonathan Caravaca, Laura Gómez, Onofre Fullana, Ivan Murray, Gabriel Jover, Xavier Cussó, Ramon Garrabou

**Affiliations:** 1 Department of Economic History, Institutions, Policy and World Economy, Universitat de Barcelona, Barcelona, Spain; 2 Department de Matemàtiques, Universitat Politècnica de Catalunya, Barcelona, Spain; 3 Department of Environment and Soil Sciences, School of Agricultural Engineering, University of Lleida, Lleida, Spain; 4 Department of Environmental Studies, Faculty of Social Studies, Masaryk University, Brno, Czech Republic; 5 Barcelona Institute of Regional and Metropolitan Studies, Autonomous University of Barcelona, Bellaterra, Spain; 6 Institute of Social Ecology, BOKU University of Natural Resources and Life Sciences, Vienna, Austria; 7 Basque Centre for Climate Change, Scientific Campus of the University of the Basque Country, Leioa, Spain; 8 Independent professional researchers, Barcelona, Spain; 9 Department of Climate Action, Food and Rural Agenda, Government of Catalonia, Barcelona, Spain; 10 Agroecosystems History Laboratory, Pablo de Olavide University, Utrera Road, Seville, Spain; 11 Department of History, College of Arts and Science, University of Saskatchewan, Saskatoon, Canada; 12 Faculty of Arts, University of Prince Edward Island, 550 University Avenue, Charlottetown, Prince Edward Island, Canada; 13 CEIGRAM Research Centre for the Management of Agricultural and Environmental Risks, Polytechnic University of Madrid, Madrid, Spain; 14 Department of Economic Theory and Economic History, Faculty of Economics and Business, University of Granada, Granada, Spain; 15 Department of Applied Economics, Faculty of Economics and Business, University of Santiago de Compostela, Santiago de Compostela, Spain; 16 Department of Social Sciences and Commerce, Marianopolis College, Westmount, Quebec Canada; 17 Institut des Sciences de la Forêt Tempérée, Université du Québec en Outaouais, Gatineau, Quebec Canada; 18 Division of Organic Farming, BOKU University of Natural Resources and Life Sciences, Vienna, Austria; 19 Department of Geography, University of the Balearic Islands, Valldemossa Road, Mallorca, Spain; 20 Department of Economics, Faculty of Economics and Business, University of Girona, Girona, Spain; 21 Department of Economics and Economic History, Economics and Business, Autonomous University of Barcelona, Bellaterra, Spain

**Keywords:** Agricultural systems, EROI (energy return on energy investment), Agroecosystem, Circularity, Socioecological transition, Dietary transition, Forest transition

## Abstract

Early energy analyses of agriculture revealed that behind higher labor and land productivity of industrial farming, there was a decrease in energy returns on energy (EROI) invested, in comparison to more traditional organic agricultural systems. Studies on recent trends show that efficiency gains in production and use of inputs have again somewhat improved energy returns. However, most of these agricultural energy studies have focused only on external inputs at the crop level, concealing the important role of internal biomass flows that livestock and forestry recirculate within agroecosystems. Here, we synthesize the results of 82 farm systems in North America and Europe from 1830 to 2012 that for the first time show the changing energy profiles of agroecosystems, including livestock and forestry, with a multi-EROI approach that accounts for the energy returns on external inputs, on internal biomass reuses, and on all inputs invested. With this historical circular bioeconomic approach, we found a general trend towards much lower external returns, little or no increases in internal returns, and almost no improvement in total returns. This “energy trap” was driven by shifts towards a growing dependence of crop production on fossil-fueled external inputs, much more intensive livestock production based on feed grains, less forestry, and a structural disintegration of agroecosystem components by increasingly linear industrial farm managements. We conclude that overcoming the energy trap requires nature-based solutions to reduce current dependence on fossil-fueled external industrial inputs and increase the circularity and complexity of agroecosystems to provide healthier diets with less animal products.

**Supplementary Information:**

The online version contains supplementary material available at 10.1007/s13593-023-00925-5.

## Introduction

This article provides an analytical synthesis of the results obtained by the international project *Sustainable Farm Systems: Long-Term Socio-Ecological Metabolism in Western Agriculture* (SFS), in which different research teams have been working since 2012 to compile the largest dataset on energy balances of past and present agroecosystems calculated so far using the same approach and methodology. The environmental history perspective of the SFS project has led us to rethink the energy accounting methods applied for half a century to mainly contemporary agricultural systems, calculating a single energy return on energy inputs (EROI) expended by farmers only considering the industrial inputs bought from outside of their farms (Pimentel et al. 1973; Leach 1975, 1976; Pimentel and Pimentel 1979; Fluck and Baird 1980; Naredo and Campos 1980; Smil et al. 1983; Stanhill 1984; Smil 1984; Dazhong and Pimentel 1984; Jones 1989; Giampietro et al. 1992; Fluck 1992; Hammerschlag 2006; Murphy et al. 2011; Pimentel 2011). Although some of these early energy case studies made comparative analyses of farming systems across countries or regions with different levels of agricultural industrialization, only one studied a nineteenth-century farm system (Bayliss-Smith 1982).

**Fig. 1:**
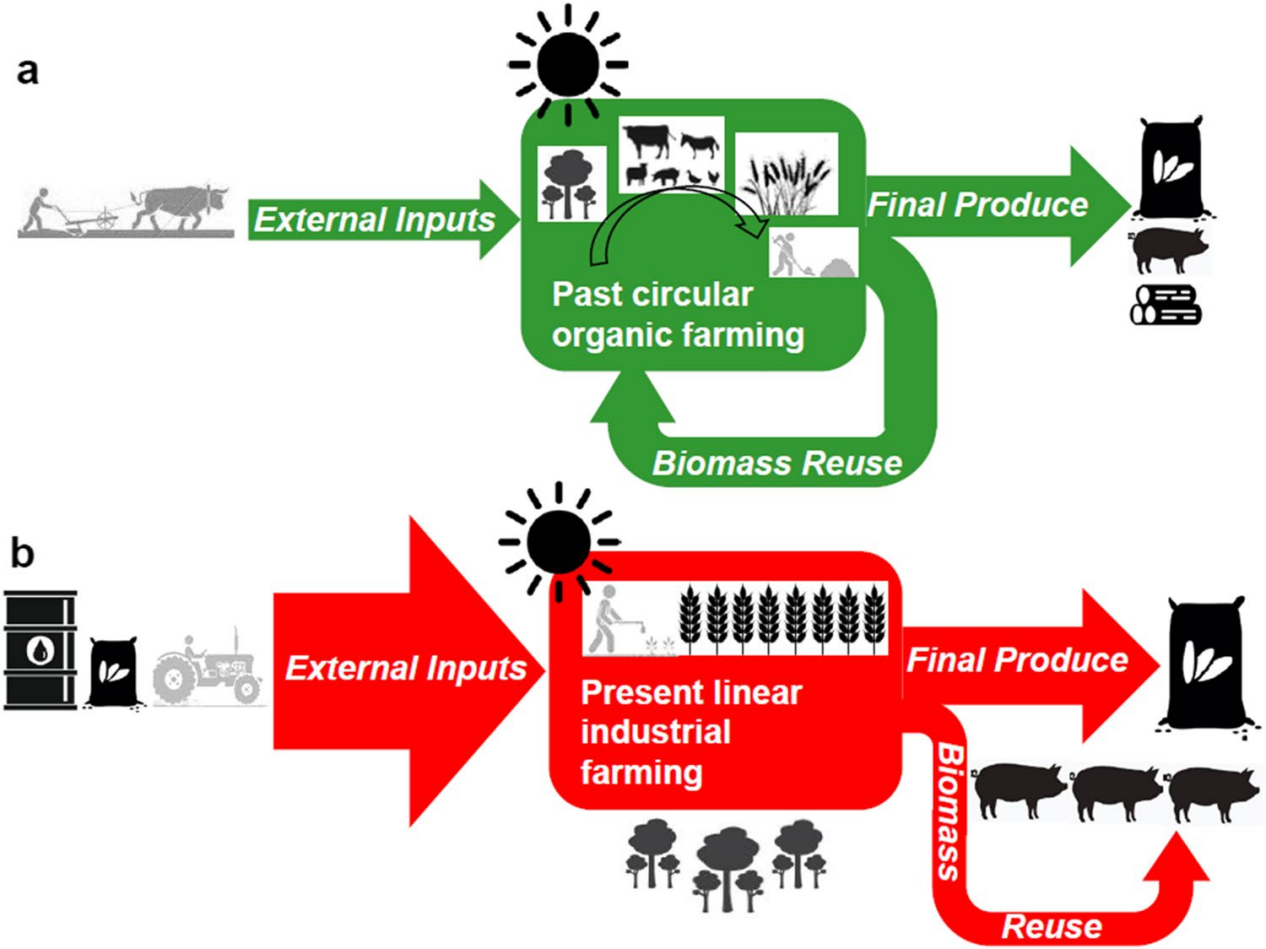
Outline of the contrasting patterns of energy flows between circular-integrated organic farming (**a**, in green) and linear-disintegrated industrial farming (**b**, in red) found in 82 agroecosystems of North America and Europe from 1830 to 2012, which explain their paths towards lower energy returns on the external inputs invested, with few or no increases in the returns on the internal inputs and on all inputs consumed. Icons for agroecosystem components (arable crops, livestock, and forests) and types of energy flows (external inputs, internal biomass reuses, and final products) are represented in black, except for the different sorts of human labor that appear in gray. Note the proportional changes in the width of the arrows representing external inputs, reuses, and final produce. Structural changes are illustrated in the direction of reuses, the composition of flows, and how the components of the agroecosystem are related or separated one another. Source: Our own.

Given the linearity of today’s industrial agriculture (Fig. 1b) that is highly dependent on external industrial inputs (seeds, synthetic fertilizers, herbicides, pesticides, tractors, electric implements, imported feed), it has made sense to focus the energy analysis on a single EROI that expresses the extent to which these farm systems are energy sinks instead of net energy suppliers to the rest of society (Marshall and Brockway 2020). This also contributes to assess what minimum EROI the societal system must achieve to maintain its own metabolic complexity (Giampietro et al. 2011, 2013). Nevertheless, to study preindustrial mainly solar-based agricultures (Wrigley 2016) means dealing with something completely different. Given the scarcity and cost of external energy sources then available, preindustrial farmers had to rely on a circular multifunctional management of their agroecosystems (Fig. 1a). Livestock played a key role in that bioeconomic circularity by being fed with cropland products (feed) and by-products (stubble, bran, husks, stalks, green shots of trees, garbage), as well as by grazing pastures and forestlands, and then recirculating its draft force and manure back to cropland (Krausmann 2004).

Cropping-pasture integration, combined with leguminous crops, was the hallmark of the English agricultural revolution in the seventeenth and eighteenth centuries and its later adoption in Atlantic and continental Europe in the nineteenth century (Campbell and Overton 1991; Allen 2008; Tello et al. 2017). Indeed, this was also a key feature of a much broader set of practices for maintaining soil fertility across continents throughout the global history of farming (McNeill and Winiwarter 2004, 2010), which the new advances towards an agroecological transition are currently recovering everywhere in the world (Gliessman 2016; Wezel et al. 2020; González de Molina and López-García 2021; Pirdashti et al. 2015; Xie et al. 2018; Farias et al. 2020).

Therefore, energy analysis of past and present farm systems can no longer conceal the role of internal biomass reuse flows of agroecosystems in an analytical black box (Tello et al. 2015, 2016; Guzmán and González de Molina 2017). These internal energy returns have two meanings. On the one hand, they account for a partial energy efficiency in the agroecosystem functioning. On the other hand, they assess the proportion of energy recirculated for the agroecosystem reproduction relative to the final product extracted. These internal matter-energy flows become temporarily stored in the living funds of the agroecosystem, such as livestock, fertile soils, and trees, while the energy extracted as products is dissipated and no longer plays a role in their sustenance. Therefore, the ratio of internal reuses compared to the energy dissipated as human consumption provides relevant information for the sustainability of agroecosystems, provided that this internal recirculation keeps a complex integration between the living funds (Fig. 1a) to prevent them from quickly becoming dissipative (Fig. 1b).

The last condition is important because societies did not always fulfill it in past times. In the expanding agricultural frontiers with a great shortage of labor relative to the abundance of land, there was often not enough labor capacity for sufficient biomass recirculation, but yields were not affected in the short term because the soils were very rich in nutrients. This was the case in the nineteenth-century North American Great Plains, where Western settlement began with cattle ranching, followed by plowing the sod for an export-oriented grain growing that was kept separate from most livestock. Only a small fraction of the nutrients removed from these soils returned to them as manure (Burke et al. 2002), and that soil mining lasted until yield decrease and population growth paved the way for greater cropping-pasture integration (Cunfer 2005, 2021; Cunfer and Krausmann 2016; Gutman 2018). Therefore, if energy analysis is to contribute to the sustainability assessment of farm systems, it must account for the energy returns on internal reuses, on external inputs, and on both at once through a multi-EROI assessment (Gingrich et al. (2018a, b, c). The last review article published on the subject in this journal considers this agroecological multi-EROI methodology the most circular energy analysis of farm systems developed to date (Hercher-Pasteur et al. 2020a, b).

Previous research on crop-specific energy balances has shown that the energy returns to external inputs were lower in highly industrialized agricultural systems than in more traditional ones, which were less dependent on industrial inputs (Pimentel and Pimentel 1979; Dazhong and Pimentel 1984; Giampietro et al. 1992). More recent research has found that efficiency gains in the production and use of agrochemicals and machinery have to some extent improved the agricultural energy returns on external inputs from the 1980s onwards (Pellegrini and Fernández 2018; Marshall and Brockway 2020), particularly in Europe (Bajan et al. 2021), although with differences between products, regions, types of management, and scales (Harchaoui and Chatzimpiros 2019; Gingrich and Krausmann 2018; Aguilera et al. 2015; Hamilton et al. 2013; Murphy et al. 2011; Pelletier et al. 2011; Dalgaard et al. 2001; Schroll 1994).

Here, we are going to answer the following research questions, aimed at advancing the energy analysis of agroecosystems using a unique set of 82 case studies of historical and current agriculture in Europe and North America: what happens when we calculate these energy balances and returns not only for specific crops, but for entire agroecosystems from past organic to current industrial agriculture? What role has the disintegration between the agricultural, livestock and forestry components of agroecosystems played in the impact of this socioecological transition on the energy performance of farming?

## Materials and methods

### Case studies

This article builds on 82 energy balances calculated in different points of time from 1830 to 2012 in 19 multi-scalar case studies of 5 countries, ranging from the farm and municipal to county or national level, always referred to whole agroecosystems encompassing cropland, pasture and forest uses, or at least two of them. These system-wide energy analyses have been carried out in Nemaha, Chase, and Decatur counties in Kansas, USA (Cunfer, Watson and MacFadyen 2018); Queens, Kings, and Prince counties in Prince Edward Island (MacFadyen and Watson 2018); the province of Quebec, Canada (Parcerisas and Dupras 2018); Sankt Florian and Grünburg villages in regions of Upper Austria (Gingrich et al. 2018a); the whole Austria (Gingrich and Krausmann 2018); Holubí Zhoř village and an organic farm in Czech Republic (Fraňková and Cattaneo 2018); seven Spanish municipalities: Santa Fe in Granada province, Andalusia (Guzmán and González de Molina 2008); Caldes de Montbui, Castellar de Vallès, Polinyà, and Sentmenat in the Vallès county of Barcelona province (Marco et al. 2018; Gómez 2017); Les Oluges in Lleida province, Catalonia (Díez-Sanjuán et al. 2018); Manacor in the Mallorca Island (Fullana et al. 2021); together with a county (Baix and Alt Maresme in Catalonia; Parcerisas, personal communication), and the whole country of Spain (Guzmán et al. 2018; González de Molina et al. 2020). The location map (Fig. SM1), the full list of case studies with the three energy returns, all other data used in the statistical analysis (Table SM1), and all values of each different energy flows considered in these 82 energy balances are in the Supplementary Material.

These case studies show differences in natural resource endowments, types of management and technical implements used, and the spatial scales of their system boundaries. Each of them has its own characteristics and history, discussed in the original articles presenting results for each case. This previous work, based on a qualitative comparison of seven regional-scale case studies, suggested an agroecosystem energy transition characterized by diverging energy profiles in traditional organic and industrial agriculture (Gingrich et al. 2018b). In this analytical synthesis, we draw on a larger panel data of multi-scalar case studies, including local, regional, and national cases, to conduct optimality analyses of the possible relationships among three interrelated EROIs compared to their actual historical shifts, and statistical analyses testing whether statistically significant trends can be identified in the changing energy profiles across this transition. If common trends appear despite their biogeographical, socioeconomic, historical differences, and the multi-scale character of the panel data, this will mean that they underwent similar structural changes that drove their long-term socioecological paths.

Traditional organic farming, as it still prevailed throughout most of the nineteenth century in Europe, relied on renewable biomass flows managed to reproduce their agroecosystem components, while agricultural colonization in North America frontiers, despite being less integrated and more extractive, also relied on very small non-renewable energy inputs (Cunfer et al. 2018; MacFadyen and Watson 2018). We denote this type as mainly solar-based farming system (Fig. 1a). In contrast, industrial agriculture as it emerged in the early twentieth century and became dominant in Western industrialized countries after the World War II (Fig. 1b) relies largely on external inputs such as synthetic fertilizers, agrochemicals for weed and disease control, machinery, and imported feed associated with high carbon emissions, water pollution, soil degradation, and biodiversity loss (Pimentel 2011; Rockström et al. 2020; Crippa et al. 2021).

### Conceptual approach to the circular energy analysis of agroecosystems

**Fig. 2:**
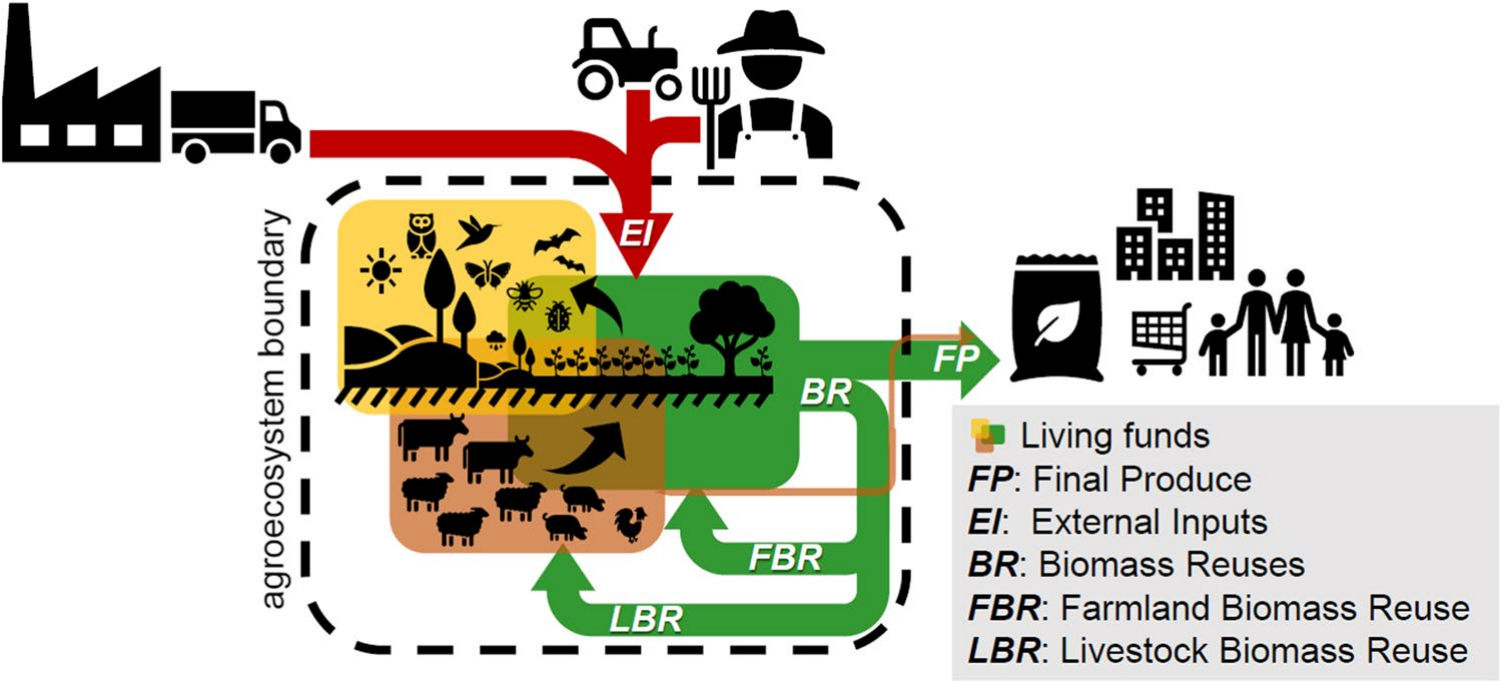
Circular approach used to account for the energy profiles of agroecosystems. Source: Our own.

Farmers build agroecosystems coproducing with nature (Gliessman and Engles 2015; Van der Ploeg 2014). Figure 2 depicts the system boundaries, the main compartments or energy “funds,” and the energy flows considered in this approach (Gingrich et al. 2018a, b, c). Our circular approach aims to highlight the structural changes between internal and external energy inputs throughout the industrialization of agriculture (Tello et al. 2016; Galán et al. 2016; Guzmán and González de Molina 2017). The conceptual frame of this agroecological multi-EROI model is the study of agricultural social metabolism—i.e., the material and energy flow accounting of the agroecosystems’ functioning—(González de Molina and Toledo 2014; González de Molina et al. 2020). The accounting methodology is based on the bioeconomic “flow-fund” analysis introduced by Georgescu-Roegen (1971, 1976) which has been further developed by Giampietro, Mayumi, and Sorman (2011, 2013).

Living “funds” are the structural components of agroecosystems that can supply a flow of useful products to farmers and society, provided their own reproductive needs are met (livestock, soils, landscapes, farm-associated biodiversity). The more diverse and integrated through internal matter-energy flows these funds are, the more complex and circular the agroecosystem is (Fig. 2). Depending on where the boundaries of the energy analysis are set, the type of products and inputs considered vary. This, combined with the adoption of a linear approach with a single EROI or a multi-EROI agroecological circular one, leads to different results expressing partial or whole system energy returns (Murphy et al. 2011; Arizpe et al. 2011; Hercher-Pasteur et al. 2020a, b). When energy analyses only consider specific crops (Pracha and Volk 2011; Pagani et al. 2017; Pellegrini and Fernández 2018), they cannot address the structural changes that industrialization of agriculture has meant for the loss of biophysical integration and circularity of agroecosystems (Patrizi et al. 2018; Marco et al. 2018; Font et al. 2020) and for landscape heterogeneity and biodiversity (Marull et al. 2019a, 2019b, 2018).

A sustainability assessment of the evolution of energy efficiency of farming must take these structural changes into account (Fig. 1), given their contribution to global warming and environmental degradation (Crippa et al. 2021; Rockström et al. 2020; Tilman et al. 2002; Tilman 1999). These detrimental impacts have a lot to do with the dependence of industrial agriculture on fossil fuel–based external inputs (Pimentel 2011), as well as with the lack of circularity and integration of agroecosystems. Reducing or overcoming dependence on external inputs will curtail environmental degradation but raises concerns about energy yields and land and labor requirements. Divesting from fossil energy inputs while improving energy returns on investment (Hammerschlag 2006) requires a new advance towards more circular agrarian bioeconomy (Schmidt et al. 2012). This agrarian bioeconomy will contribute to the Sustainable Development Goals as proposed by the UN Committee on World Food Security (CFS 2021; Caron et al. 2018), the FAO 2018 Scaling Up Agroecology Initiative (FAO 2018), the IPCC (2019) recommendations in the special report on Climate Change and Land, and the new EU agroecology initiatives beyond the Farm to Fork Strategy within the European Green Deal (European Commission 2022).

### The circular multi-EROI accounting method of agroecosystems

The differentiation between external inputs and recirculation of internal biomass flows is the cornerstone of our circular bioeconomic approach that combines three EROI indicators to analyze the changing flow-fund patterns of agroecosystems (Table 1).

**Table 1:** Agroecosystem living funds and energy flows considered in the multi-EROI approach. Source: Our own (from Tello et al. 2015, 2016). *This requires accounting for the actual net primary production (NPP) of the agroecosystem to then subtract human appropriation and obtain the fraction of unharvested phytomass directly taken by wildlife, which is not included in this article (Guzmán and González de Molina 2017). **Animal traction force and manures are not added as energy inputs to the livestock feed to avoid double counting.

Funds & main flows	Main components of each fund & flow
Living funds of an agroecosystem (they provide flows if their own reproduction needs are met)	Farmland: all annual herbaceous crops & perennial arboriculture
	Uncultivated land: woods, brushwood & grasslands
	Livestock: breeding cattle & other, with working animals if applicable
	Biodiversity: non-domesticated flora & fauna in agroecosystems*:
	belowground (soil biota)
	aboveground (in soil covers)
External inputs (*EI*): their energy content + embodied energy required to produce & transport them + energy amortization (if applicable)	Farm labor: the share of farmers food intake required to work
	Farming community inputs: domestic garbage, humanure if applicable
	Industrial inputs:
	Seeds bought from outside with transport & embodied energy
	Feed bought from outside with transport & embodied energy
	Tractors & farm implements with fuel & embodied energy
	Synthetic fertilizers with transport & embodied energy
	Pesticides & herbicides with transport & embodied energy
	Electricity (pumps, heaters…) & embodied energy
Internal biomass reuses (*BR*): their energy content (up to animal feeding)	*FBR*: Biomass Reuse flows that go directly to cultivated soils or sown meadows:
	Seeds selected and reused inside the agroecosystem
	Green manures
	Pruning, leaves, stubble & another biomass buried fresh or burnt
	*LBR*: Biomass reuse flows through livestock:
	Livestock grazing & feed**

Based on this multi-EROI accounting method explained in Tello et al. (2016), we calculated three different and interrelated energy indicators using as output the useful biomass provided to farmers and society at the exit gate of the agroecosystem considered (*FP* or *final produce*). The most aggregate EROI indicator is the *final EROI* (or *FEROI*), which measures the energy return in terms of the ratio of *FP* biomass flows to the whole set of energy carriers used as inputs, either coming from outside or within the agroecosystem (*TIC or total inputs consumed*):

1 $$Final\;EROI\;\left(or\;FEROI\right)=\frac{Final\;Produce\;\left(FP\right)}{Total\;Inputs\;Consumed\;\left(TIC\right)}.$$

*TIC* can be split into *external inputs* (*EI*) and the internal flows of *biomass reused* (*BR*), where $TIC = BR+EI$. This allows to decompose *FEROI* into two other energy indicators, the

2 $$External\;Final\;EROI\left(\mathrm{or}\;EFEROI=\frac{Final\;Produce\;\left(FP\right)}{External\;Inputs\;\left(EI\right)}\right)$$

and the

3 $$Internal\;Final\;EROI\;\left(\mathrm{or}\;IFEROI=\frac{Final\;Produce\;(FP)}{Biomass\;Reused\;(BR)}\right)$$

Distinguishing between *BR* and *EI* flows, and accounting for them in a systemic way, provides a consistent analysis of the long-term $\frac{EI}{BR}$ structural shifts. Recall that *IFEROI* is not only a partial indicator of energy efficiency, but also the ratio of the biomass energy reinvested in the reproduction of the agroecosystem living funds to the *FP* dissipative energy extracted from it. The core idea underpinning this conceptual approach is the principle that all living systems rely on internal biophysical cycles that sustain their reproduction over time (Jordan 2016). The recirculation of matter-energy flows allows them to maintain complexity, increase temporary energy storage, and decrease internal entropy by keeping them away from thermodynamic equilibrium that means death (Ho 2013; Morowitz and Smith 2007). All this also applies to agroecosystems (Gliessman and Engles 2015; Guzmán and González de Molina 2017).

### Analyzing the changing energy profiles of agroecosystems along socioecological transitions

To identify general trends in the changing energy profiles of agroecosystems, we use the following function that relates *FEROI*, *EFEROI*, and *IFEROI *values (Tello et al. 2016):

4 $$FEROI = \frac{EFEROI\cdot IFEROI}{EFEROI+IFEROI}$$

The proof is straightforward: $\frac{EFEROI \cdot IFEROI}{EFEROI+IFEROI}\;=\;\frac{\frac{FP}{EI}\;\cdot\;\frac{FP}{BR}}{\frac{FP}{EI}+\;\frac{FP\;}{BR}}=\;\;\frac{\frac{{FP}^{2}}{EI \cdot BR}\;}{\frac{FP(EI+BR)}{EI \cdot BR}\;}\;=\;\;\frac{FP}{EI+BR}\;=\;FEROI$.

**Fig. 3:**
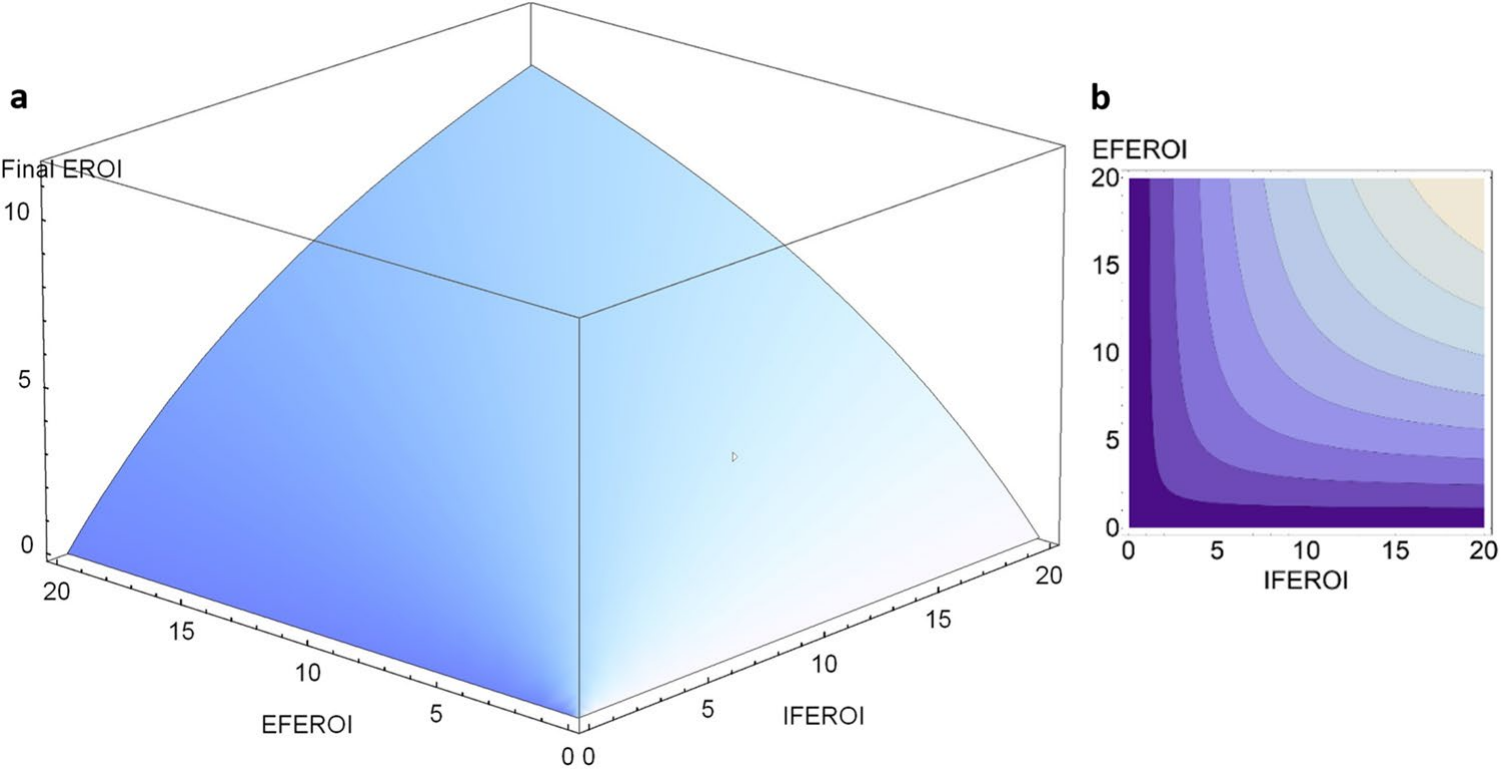
**a** Graphical representation of all possible values of final energy returns on all inputs invested $\left(FEROI=\frac{Final \;Produce\; (FP)}{External \;Inputs \;\left(EI\right)\;+\;Biomass \;Reuses \;(BR)}\right)$ as a function of final energy return on external inputs $\left(EFEROI = \frac{Final \;Produce\; (FP)}{External \;Inputs \;(EI)}\right)$ and final energy return on internal reuses $\left(IFEROI = \frac{Final \;Produce\; (FP)}{Biomass \;Reuses \;(EI)}\right)$ shown in a tridimensional figure. **b** The same, shown in a bidimensional energy map where contour lines represent the third *FEROI* dimension. Source: Our own, by Vera Sacristán from Tello et al. (2016).

Expression (4) is the equation of a three-dimensional surface that encompasses all the values that *FEROI*, *EFEROI*, and *IFEROI* can take at the same time (Fig. 3a).

In any visualization of empirical results, this surface is limited by the highest EROI value found in the analyzed agroecosystems, since despite the increasing curvature of the surface towards the vertical axis it does not have a theoretical upper limit. The curvature reveals the existence of diminishing returns at any point (i.e., additional *FEROI* increases always require greater proportional increases in *EFEROI*, *IFEROI*, or both). In Fig. 3b, this possibility surface is drawn as a two-dimensional “energy map” where the contour levels represent equal *FEROI* values.

As these energy maps show the three possible EROI values of an agroecosystem at the same time, they can visualize the changing energy profiles of farm systems throughout the socioecological transition from preindustrial organic to full industrial agricultures in a deeper analytical way than using three time series for each EROI, as was done before based on a limited number of case studies (Gingrich et al. 2018b; see Figs. SM2, SM3 and SM4 in the Supplementary Material). High *EFEROI* values would be the hallmark of traditional solar-based organic agriculture due to their low dependence on external inputs, which in turn would require a great reliance on internal recirculation of biomass flows and lower *IFEROI* values. Accordingly, the *FEROI-IFEROI-EFEROI* coordinates of traditional organic agroecosystems would be near the upper left corner in the energy map (Fig. 3b). Industrialization would free agricultural systems from labor-intensive biomass reuses by replacing them with increasingly cheaper external inputs based on fossil fuels, moving their energy profiles towards other regions of the energy map. Any displacement along the contour lines expresses a change in the energy profiles of agroecosystems in terms of their *EFEROI*-*IFEROI* values while keeping the same value level of *FEROI*, whereas any displacement outside contour lines also means *FEROI* increases or decreases.

**Fig. 4:**
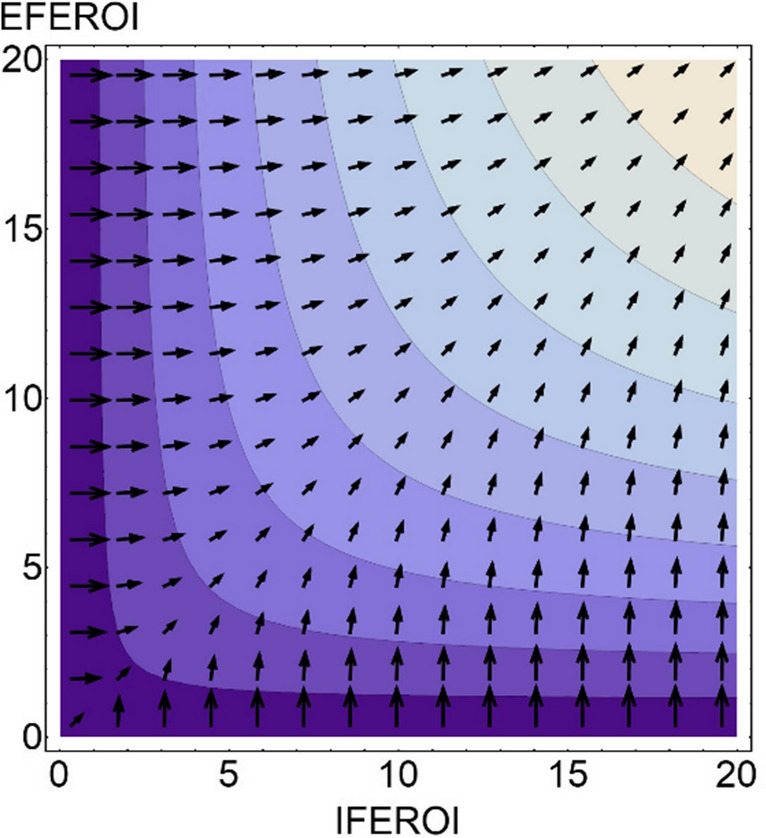
Gradient-vector field indicating optimal paths towards improvement of final energy returns on all inputs invested $\left(FEROI=\frac{Final \;Produce\; (FP)}{External \;Inputs\; \left(EI\right)\;+\;Biomass\; Reuses \;(BR)}\right)$ playing with $\frac{Final\; Produce\; (FP)}{External \;Inpunts\; (EI)}$ and $\frac{Final \;Produce\; (FP)}{Biomass\; Reuse \;(BR)}$ shifts at any point of the possibility surface. Source: Our own, by Vera Sacristán. The demonstration of the calculation used with Eq. (4) is in the annex of Tello et al. (2016). The values of the vertical axis (final energy return on external inputs or EFEROI), of the horizontal axis (final energy return on internal reuses or IFEROI), and of the contour lines (final energy return on all inputs invested or FEROI), are the same as Fig. 3b.

This possibility surface allows to calculate optimal shifts to increase *FEROI* scores by changing the *EFEROI*-*IFEROI* variables (Fig. 4), another useful reference to compare with the actual paths. Note that the gradient direction of each vector expresses, at any point of the energy map, the optimal *EFEROI*-*IFEROI* value shifts required to attain the largest possible *FEROI* increase there. The length of each vector expresses the respective potential of *FEROI* improvement for any agroecosystem placed in different regions of the energy map. The shorter length of vectors as they move towards higher *FEROI* values indicates the inevitable diminishing returns due to entropy.

This is a descriptive analysis, not a prescription. We know that higher *FEROI* values are beneficial to farmers, and to society at large, but only if all else remain equal or better. Since we cannot take this for granted, more research is required on the impacts of these energy changes on different dimensions not included in the model to consider potential trade-offs. However, comparing the real *FEROI*-*EFEROI-IFEROI* paths with the optimal ones provides a useful information to interpret the changing energy profiles of agroecosystem throughout socioecological transitions. Here, we use for the first time this multi-EROI possibility surface as an energy mapping of the changing energy profiles of agroecosystems from past organic to current industrial management.

### Statistical analyses of the main drivers of *FEROI, EFEROI*, and *IFEROI* trends

Historical studies of our 82 energy balances performed one by one suggested the hypothesis that the main drivers of long-term *FEROI-EFEROI-IFEROI* trends may have been the changing role of cropping, livestock raising, and forestry along the structural change from the organic farming of preindustrial times, highly circular and integrated, to the highly linear and disintegrated current industrial agriculture (Fig. 1).

To test this hypothesis, we used linear mixed-effects models in the panel data of these 82 energy balances with either *FEROI*, *EFEROI*, or *IFEROI* as dependent variables, introducing as fixed effects the spatial scale (*S*) of the case study (i.e., farm, village, county, province, country), the year to which each energy balance corresponds (*Y*), the final energy product per unit of farmland area (*FP*), the human labor performed in energy terms per farmland area (*L*), the relation between woodland and farmland area (*WS*), the livestock energy produce per farmland area (*LV*), and the proportions of final product obtained from woodland (*W_FP*) and from livestock (*LV_FP*). Each case study was introduced as a random effect nested within its country. *FP* and *L* are used as control variables for natural resource endowment, land use intensification, and technical change, which are needed given the large differences between the case studies in these respects. Introducing *Y* as independent variable avoids temporal autocorrelation, and introducing the random effect avoids spatial autocorrelation. The analysis was performed with the package “Rcmdr” (Fox 2005) in R (R Development Core Team 2009). Models were chosen that complied with basic statistical assumptions, including the absence of multicollinearity, and that improved the AIC value by at least two units in relation to the other models. When necessary, response variables were transformed, or influential values were removed from the data.

We performed an additional test, shown in the Supplementary Material, to search for statistically significant differences among the three periods studied: traditional organic (1830–1900), intermediate organic-industrial (1901–1950), and full industrial agriculture (1951–2012). Paired sample *t*-tests with a significance level of 0.05 were run between pairs of the three periods. When multiple years were available for a case study in any given period, we kept only one value by removing the values for those years closest to the other periods. These three statistical tests of linear mixed effects, and the additional paired sample *t*-test, provide much stronger insight into the underlying driving forces of the main common trends in the observed muti-EROIs, compared to the previous summary with only one part of this database published in Gingrich et al. (2018b).

## Results and discussion

### The energy trap of industrial farming

**Fig. 5:**
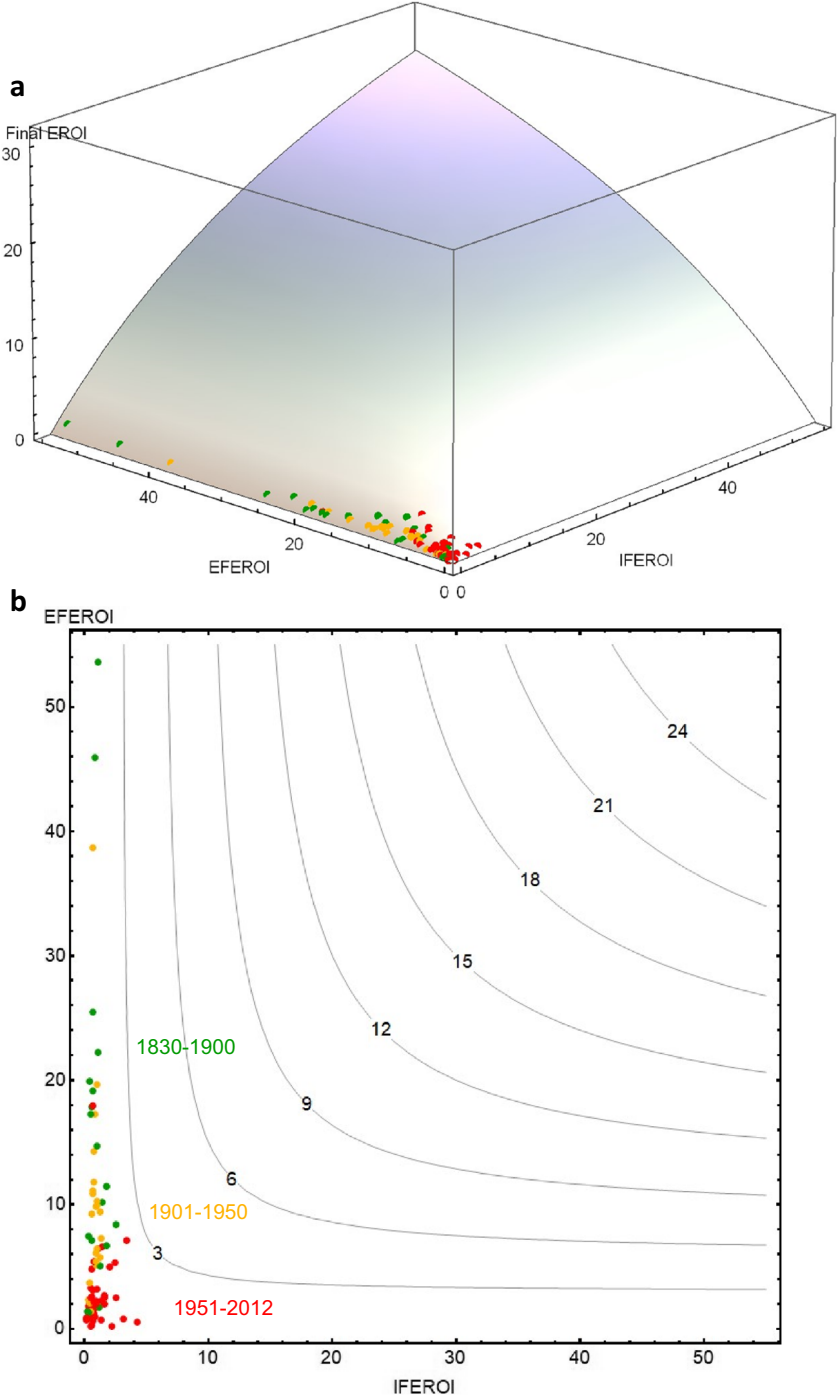
**a** Energy returns on investment (EROI) results of the 82 agroecosystems studied (in green dots, past organic, in yellow dots intermediate organic-industrial, in red dots full industrial) plotted in the possibility surface of all the values that final energy returns on all inputs (*FEROI* in the vertical axis), final energy returns on external inputs (*EFEROI* in the left horizontal axis), and the final return on internal reuses (*IFEROI* in the right horizontal axis) can jointly take according to Eq. (4) of the article. **b** The same, shown in a bidimensional energy map where *FEROI* values are shown in the contour lines. Source: Our own, by Vera Sacristán from the data shown in Table SM1 and the Excel file of the Supplementary Material.

Figure 5 depicts the panel data of 82 farm systems as points with different color according to the historical period in the above three-dimensional possibility surface. Below the figure depicts the same results in the bidimensional energy map where *FEROI* values are shown with contour lines.

The changing energy profile of our 82 agroecosystems displays a general trend that we name an “energy trap.” This energy trap is defined as the clustering of most *FEROI-EFEROI-IFEROI* industrial farming data near to the origin axes of the three-dimensional surface encompassing all possible values these three EROIs can simultaneously take. In 16 out of 19 case studies, energy returns on external inputs (*EFEROI*) were higher in the traditional organic group than in the industrial farming group. In the industrial group, the energy returns on internal biomass flows (*IFEROI*) were greater than in the traditional organic cases in 15 cases, but these *IFEROI* increases are smaller than the corresponding *EFEROI* decreases (see also Table SM1 and Fig. SM5 in the Supplementary Material). As a result, in this dataset, we do not see cases displaced to the right corner of Fig. 5 with very high *IFEROI* values. Finally, the *FEROI* values are lower in the industrialized farm systems than in the traditional organic ones in 12 of the 19 case studies, but the decreases are again smaller than those experienced by the *EFEROI* values. These simultaneous *FEROI-EFEROI-IFEROI* changes driven by increases in external inputs (*EI*) greater than the corresponding increases in final product (*FP*), and greater than decreases in biomass reuses (*BR*) when they occurred, has brought their energy profiles closer to the origin vertex of the energy map where the values of the three EROIs are the lowest (Fig. 5). Therefore, our answer to the first research question is that agricultural industrialization has led to an energy trap when external, internal, and total input returns are considered together in a long-term historical perspective for entire agroecosystems, and not only single crops or activities.

The general picture of the energy trap of industrial farm systems shown in Fig. 5 is confirmed by the basic statistics of the *FEROI-EFEROI-IFEROI* dataset (see Table SM3 and the Excel file in the Supplementary Material). According to the paired-samples *t*-tests, *IFEROI* values were not significantly different (*p*-value = 0.15) from traditional organic (1830–1900) to intermediate organic-industrial farming (1901–1950), but differences were close to significance for *EFEROI* (*p* = 0.052) and for *FEROI* (*p* = 0.07). This confirms that the main structural changes in the flow-fund patterns of agroecosystems (Fig. 1) took place with the full industrialization of farming after the World War II. From the intermediate organic-industrial (1900–1950) to full industrial farming (1951–2012), the difference is significant for *EFEROI* (*p* = 0.004) and *IFEROI* (*p* = 0.007), as it is from traditional organic (1830–1900) to full industrial farming (1951–2012) for *EFEROI* (*p* = 0.003) and *IFEROI* (*p* = 0.04) as well, but not for *FEROI* in both cases. This confirms that the higher dependence on fossil-fueled external inputs (*EI*) went hand in hand with lower efforts in biomass-energy reinvestment (*BR*) in the reproduction of the living funds of the agroecosystems from the 1950 onwards. Conversely, the much lower reliance on *EI* of past organic farming involved higher *BR* values per unit of final produce (*FP*).

Our corroboration of the long-term energy trap of industrial agriculture contrasts with the results obtained in several studies, including some of our SFS project, which have found improvements in external EROIs (i.e., *EFEROI* here) of industrial farming from the 1980–1990s onwards (Marshall and Brockway 2020; Harchaoui and Chatzimpiros 2019; Pellegrini and Fernández 2018; Gingrich and Krausmann 2018; Aguilera et al. 2015). The long-term historical character of our dataset puts these later results into clearer perspective. The improvements observed in recent decades exist but are very small compared to the steep *EFEROI* decline during the transition from traditional solar-based to current fossil-based agriculture.

The mean *FEROI* values were not significantly different from traditional organic to full industrial farming time periods due to 7 cases of full industrial farming that outperform those of traditional organic or intermediate organic-industrial systems (Fig. 5, and Supplementary Material). This can be explained by the different composition of their agroecosystems and the way they changed over time. Two of them are in the Great Plains of the USA where colonization began in the 1870–1880s through extensive cattle ranching with extremely low *IFEROI* and *FEROI* values, placing their green dots near to the origin vertex in the bottom corner of Fig. 5. They then evolved into an intermediate organic-industrial mixed farming more integrated with pasture and higher *FEROI* values, until the shocks of the Great Depression and the Dust Bowl drought in the1930s that led to an early adoption of industrial agriculture compared to Europe. This, in turn, gave rise to either higher (Nemaha and Decatur) or lower (Chase) *FEROI* values in 1954 also depending on variations in rainfall, soil quality, and proportion of livestock raising (Cunfer et al. 2018; Cunfer and Krausmann 2016; Cunfer 2005).

Other exceptions with *FEROI* industrial values greater than those of traditional organic or intermediate organic-industrial agricultures were in colder and wetter bioregions such as the Canadian Prince Edward Island (McFadyen and Watson 2018). There, the importance of forest products leveled out higher energy returns in the long run, except when cereals, potatoes, and livestock became more important and decreased *EFEROI* scores (Queens County). In the Czech village of Holubí Zhoř, the *FEROI* and *IEFROI* values of traditional organic farming were scant due to the cost of livestock feeding in the poor soils of the Bohemian-Moravian highlands with low temperatures and rainfall, compared to a current organic farm in this place (Fraňková and Cattaneo 2018). In Sankt Florian municipality of Upper Austria, a cropland specialization of rich soils meant industrial higher *FEROI* values (including the sale of straw, a flow currently reused or wasted in other places), compared to traditional organic farming when livestock densities were similar but meant a higher energy burden (Gingrich et al. 2018a). This later shift went contrary to the one found in the neighboring Grünburg municipality, specialized on cattle and pig rearing, as well as in the whole of Austria despite the rise in *FEROI* values in 1991 and 2010 (Gingrich and Krausmann 2018).

Therefore, upon closer examination, these exceptions have a lot to do with the agroecosystem composition and economic specialization (Gingrich et al. 2018b) making their paths consistent with the interpretation of the main drivers behind the general trend towards the energy trap: the change of livestock and forestry components were the main explanation of these different cases, together with land and labor endowments. All in all, these cases remind us that the overall trajectory toward steeply decreasing *EFEROI* scores, combined with only minor *IFEROI* increases and almost no *FEROI* improvements, was not a necessity but a historically contingent result of a global, but regionally differentiated socioecological transition. The fact that some common trends appear despite the large differences among these 82 agroecosystems indicates that they shared certain structural changes that drove their long-term paths.

### Structural changes: livestock and forestry roles in the energy transition

The results of the mixed-effects models confirm that the growing relevance of livestock production and the declining relevance of forestry have been two main drivers of the *FEROI-IFEROI-EFEROI* values adopted during the transition from traditional organic to full industrial farm systems in the Global North countries, counties, and municipalities of our dataset. They were decisive factors that drove the profiles of energy returns on all inputs consumed, on internal biomass reuses, and on external inputs in the 82 agroecosystems of the panel data, once the differences in natural resource endowment and land and labor intensities have been controlled, as well as temporal and spatial autocorrelation. *FEROI* values increase with *FP* per unit of land and with woodland share in the farmland area (*WS*), whereas they decrease as human labor (*L*) per unit of land and the livestock produce share in the final product (*LV_FP*) increase, as expected. This is shown in the mixed-effects model (5) where log (*FEROI*) values also significantly decrease as the year (*Y*) of the energy balance is more contemporary:

5 $$\log\;\left(FEROI\right)=2.71+0.01\cdot FP-2.65\cdot LV\_FP+1.66\cdot WS-0.28\cdot L-0.002\cdot Y$$

Among all the variables that have a significant effect on log (*FEROI*), the ones with the greatest weight are *LV_FP*, *WS*, and *FP*, in this order. AIC values for the chosen models and their null models and chi sq. and *p* (>chi sq.) values for each variable are given in the Supplementary Material for all the three mixed-effects models.

Converting log (*IFEROI)* into the dependent variable gives the following Eq. (6), where energy yields as control variable (*FP*) have a higher weight than the relevance of woodland in the farmland area (*WS*):

6 $$\log\;(IFEROI)=-1.13+0.02\cdot FP+1.65\cdot WS$$

This result confirms a feature already observed in Gingrich et al. (2018b). On the one side, the variation in the relevance of woodland share (*WS*) is significant given that forestry entails a much higher energy *FP* with any *BR* per unit of land. On the other side, the predominant *BR* trends per unit of land found in the dataset (see the Supplementary Material) are the maintenance of internal biomass reuse flows (*BR*) devoted to livestock feeding or too slight a decrease of them, which turn *LV* share in *FP* statistically not significant here. However, we know that behind those steady trends in livestock-related *BR* flows, there has been a profound structural change from mixed organic farming, where extensive grazing integrated all land uses with each other, to livestock feeding in linear industrial feedlots disintegrated from the rest of farmland (Fig. 1). This feature is clearly observed using the entire energy balance as a scanning of the underlying structural flow-fund pattern of most case studies (see the Excel file in the Supplementary Material).

Regarding *EFEROI*, we removed the 2012 balance of the Czech Republic of a single organic farm because it was an influential value, and we also used log (*EFEROI*) as dependent variable to obtain statistically significant results in Eq. (7):

7 $$\log\;\left(EFEROI\right)=19.65-0.01\cdot Y+1.38\cdot W\_FP-2.82\cdot LV\_FP-0.50\cdot L$$

The variable with the most important effect is the year of the balance sheet (*Y*) so that when the year is more recent, the lower is *EFEROI*. This clearly confirms the energy trap of industrial agriculture driven by increases of external energy inputs (*EI*) greater than the growth in the final energy produce (*FP*) obtained. The second most significant drivers are the share of the final energy product obtained from woodland (*W_FP*) with a positive effect and that obtained from livestock (*LV_FP*) with a negative effect. This confirms the importance for the energy trap of the reduction or abandonment of forestry in most places of the Global North, together with the dietary transition to a greater production and consumption of meat. And then, finally, the control variable of labor intensity (*L)* appears significative with the negative sign as expected.

According to these results, in addition to the increasing expenditure of fossil-fueled agrochemicals and machinery in *EI* values, the two main factors that most explain the multi-EROI variation of these 82 agroecosystems in North America and Europe are the decreasing proportion of forestry and the growing proportion of livestock in the final energy produce. Taken together, they mean that industrialization of farming has deeply changed the energy profiles of the flow-fund patterns of agricultural systems (Fig. 1). In most cases, synthetic fertilizers accounted for the largest share of external energy inputs (*EI*), greater than machinery and fuel (Aguilera et al. 2015). Once farmers were able to replenish soil fertility with cost efficient fossil-based fertilizers, they no longer needed to rely on either livestock manure or biomass transfers between agroecosystem compartments to replenish depleted cropland soils, breaking the energy-nutrient nexus between crops, livestock, and grazing land that was key to traditional organic agriculture (Krausmann 2004). The end of the multipurpose use of livestock as recycler of crop by-products, provider of manure and driving force, and carrier of soil nutrients from uncultivated to cultivated land has meant a structural change of agroecosystems led by the nutritional transition towards a diet with very high meat and dairy consumption in the Western countries here studied (Schramski et al. 2020; Henry et al. 2019; Alexander et al. 2016; Westhoek et al. 2014).

Worldwide, the share of crops allocated to livestock feeding grew from 10 to 45% of global production of grains throughout the twentieth century (Haberl et al. 2016; Smil 2000). In Spain, the energy content of land produce diverted to livestock feeding rose from 28% in 1900 to 53% in 2008 (Guzmán et al. 2018). While livestock was managed at the service of cropland for millennia, current industrial agriculture cultivates a large amount of land at the service of livestock with great matter-energy losses due to this inefficient animal bioconversion of grains that could provide food for a greater number of humans (Alexander et al. 2017). This explains why, instead of a simple substitution of *EI* for *BR*, agricultural industrialization entailed a functional change that turned *BR* flows into feed and fodder while reducing or abandoning pastures and the reuse of crop by-products as animal feeding (Soto et al. 2016; Marco et al. 2018; González de Molina et al. 2020). The growth of cultivated feed has countered the simultaneous abandonment of other traditional forms of biomass recirculation, such as green manures, composting of animal manure, and crop rotation with legumes (Fig. 1). Despite the substitution of tractors for horses and mules, the number of cattle, pigs, and hens has greatly increased livestock densities only to produce animal protein. In some industrial farm systems with a high share of animal production, imported feed becomes the largest external input (Padró et al. 2017; Díez-Sanjuán et al. 2018).

In traditional solar-based agroecosystems, the high land and energy costs of livestock feeding were addressed through a close integration of animal husbandry with complex land uses (Patrizi et al. 2018; Guzmán et al. 2011; Guzmán and González de Molina 2009). This integrative role has virtually disappeared with livestock industrialization. Current feedlots perform a linear feed-to-meat bioconversion disconnected from the rest of the agroecosystem living funds (Fig. 1). Therefore, in addition to the steep increases in external inputs (*EI*), our results show that blundering into the energy trap has to do with the structural change of agroecosystems in the relationship between farmland and livestock that has limited or totally offset the *BR* decreases while deeply modifying its role (Marco et al. 2018).

It helps realize the energetic importance of this disintegration to compare the partial returns of organic-multifunctional and industrial livestock raising using either a circular integrated accounting or a linear one. When the linear energy yield of a feed-to-meat bioconversion is accounted for at the barnyard or feedlot gate, industrial livestock breeding outperforms traditional multifunctional animal husbandry—although at the expense of animal wellbeing and pollution problems from the disposal of too much manure slurry than nearby farmland can absorb. When compared with an agroecosystem circular way, either traditional organic or novel agroecology managements outperform the industrial feedlots due to the addition of manure and driving force as outputs, and the reuse of by-products as input savings (Marco et al. 2018; Patrizi et al. 2018; Tello et al. 2016; Pérez-Neira et al. 2014; Pirdashti et al. 2015).

The disintegration between livestock and the entirety of agroecosystems has also put an end to the previous balance kept on livestock size relative to cropland and forest components (Fig. 1). This, and the increase in world feed trade, has led to quantities of manure that exceed the capacity of nearby cropland to absorb them in importing regions with high livestock densities, turning slurry into a polluting waste (Cattaneo et al. 2018). Meanwhile, soil organic matter is being depleted in feed exporting regions (Padró et al. 2017, 2019; Infante-Amate et al. 2022). Both contribute to breaking the global N and P biogeochemical cycles on which soil fertility depends (Rockström et al. 2020; Billen et al. 2021).

The decline of forestry and agroforestry, and the consequent shrinking relevance of wood biomass in agricultural produce (*FP*), is the second structural change that drove the energy trap of industrial agriculture by disintegrating forests from the rest of agroecosystem living funds (Fig. 1). Wood is the densest energy carrier of all biomass products that can be gathered in large quantities with comparatively less effort. The diminishing importance of wood in many parts of the global North has gone hand in hand with the land-sparing effect of an increasingly intensified agriculture segregated from forest uses (Gingrich et al. 2007). In Spain, the share of wood in the agricultural output halved from 1950 to 2010 (Soto et al. 2016), which resulted in lower *EFEROI* and *FEROI* values (Guzmán et al. 2018). Conversely, forestry intensification (e.g., in some parts in the Canadian Prince Edward Island) contributed to relatively higher *FEROI* because forestry uses less *EI* per unit of *FP* than cropland, and almost no *BR* at all. Forest transition, consisting of a decreasing importance of wood in many of our case studies, led to lower final energy returns (*FEROI*) and reinforced the decrease of external returns (*EFEROI*) as well.

### On the ways out from de energy trap of industrial agriculture

Our interpretation of the statistical results is confirmed when we closely examine in the 82 energy balances how the living funds of agroecosystems are interconnected by their matter-energy flows, and we discover a loss of biophysical circularity and complexity in most industrial cases (Marco et al. 2018; Font et al. 2020). This also suggests that the same factors underlying the poor energy performance of industrial agriculture have also led to severe and manifold environmental degradations (Rockström et al. 2020; Crippa et al. 2021; Tilman et al. 2002). Could this degradation of agroecosystems have been an additional cause of the energy trap of industrial agriculture? If this reversal causation holds true, industrial farming would have involved an eco-inefficient endeavor to substitute external inputs (*EI*) for internal functioning of natural processes (*BR*), both belowground through the turnover of organic matter that feeds soil biota and sustains its fertility (Maeder et al. 2002) and aboveground in the land cover complexity that hosts all kinds of biodiversity-related ecosystem services (Carpenter et al. 2009; Duru et al. 2015; Marull et al. 2019a). Degrading the nature-based ecosystem services has compelled industrial farmers to replace them by increasing amounts of non-renewable external inputs of mechanical and agrochemical character (Giampietro 1997).

This hypothesis is also supported by other research showing that the biophysical yield gaps between organic and industrial farming at the crop level (Ponisio et al. 2015; Pagani et al. 2017) can be compensated for by the higher landscape agroecological synergies that characterized the circular bioeconomy of many traditional organic farming and are now being recovered by new agroecology farm managements (Padró et al. 2017, 2019, 2020; Wezel et al. 2020). Addressing this question requires forthcoming research combining energy analysis with other assessments, such as soil nutrient balances (Tello et al. 2012; González de Molina et al. 2015; Gingrich et al. 2015; Cunfer 2021; Galán 2021; Güldner 2021; Corbacho and Padró 2021; Larsen 2021; Güldner et al. 2021), energy-landscape integrated analyses (Marull et al. 2019b, 2018), and other modeling from a nexus approach (Alexander et al. 2015; Giampietro et al. 2011, 2013). To that aim, the agroecological multi-EROI model here summarized is a first step in the research needed to advance towards more sustainable and circular agrifood systems within planetary boundaries (Tello and González de Molina 2017, 2023).

**Fig. 6:**
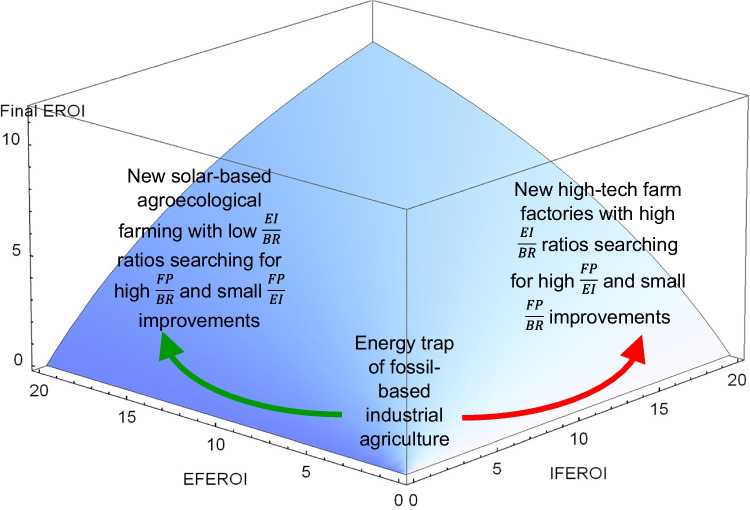
Two way-out options to the energy trap according to the multi-EROI optimality analysis of farm systems shown in Fig. 4. The green arrow to the left denotes organic-agroecological paths, and the red arrow to the right denotes the paths of industrial farm factories*.* Notice that on the left side of the graph, where organic-agroecology farming is placed, the final energy returns on external inputs $\left(EFEROI = \frac{Final \;Produce \;(FP)}{External \;Inputs\; (EI)}\right)$ are higher than the final energy returns on internal reuses $\left(IFEROI = \frac{Final \;Produce \;(FP)}{Biomass\; Reuses\; (EI)}\right),$ and the $\frac{External \;Inputs \;(EI)}{Biomass\; Reuses\; (BR)}$ ratio is lower than one, so that improvements of final energy returns on all inputs $\left(FEROI=\frac{Final \;Produce (FP)}{External \;Inputs\; \left(EI\right)\;+\;Biomass \;Reuses\; (BR)}\right)$ depend mostly on getting higher *IFEROI* returns. On the right side, where industrial farm factories are placed, it is the opposite. Most of the *FEROI* improvement depends on getting higher *EFEROI* returns because biomass reuses are lower than external inputs, and the $\frac{EI}{BR}$ ratio is higher than one, so that further *IFEROI* increases become almost irrelevant. Source: Our own.

The multi-EROI optimization analysis explained above can also be useful in forthcoming research to identify and compare the existing options to overcome the energy trap of fossil fuel-based industrial agriculture. According to the directions and lengths of the gradient vectors to improve the final energy returns of farm systems (*FEROI*) by changing their internal and external energy returns (Fig. 3b), two main roadmaps can be discerned: on the one hand, towards a new agroecology transition aimed at overcoming the current dependence on external inputs through the search for higher final energy returns from nature-based solutions relying on the internal recirculation of biomass within closely integrated landscapes and territories or, on the other hand, towards new industrial farms such as high-tech greenhouses and vertical crops relying on a higher consumption of renewable energy while saving on land and internal recirculation of biomass (Fig. 6).

The shift towards the left upper agroecological region in Fig. 6 points to a sustainable way-out based on increasing $\frac{FP}{BR}$ energy returns (*IFEROI*), by reintegrating the living funds of agroecosystems into more complex and bio-economically circular food territories (Altieri and Nicholls 2012; González de Molina and López-García 2021). According to our analysis, restoring sustainable forestry and agroforestry to abandoned woodland in the Global North, reducing livestock production and consumption, and restarting extensive livestock grazing that reintegrates forests, grasslands, and cropland management would drive such agroecological advances that increase *IFEROI* and *FEROI* returns. This fits with current prospective scenarios of a European agroecology transition (Poux and Aubert 2018; Billen et al. 2021; European Commission 2022), in line with FAO (2018) and with United Nations proposals (CFS 2021).

Conversely, agricultural factories located in the opposite bottom right region of the same Fig. 6 might also try to replace fossil synthetic fertilizers with compost, stop using pesticides, and increase $\frac{FP}{EI}$ returns (*EFEROI*) through self-production of renewable energy. However, like any other factory, these would no longer be agroecosystems but industrial sites. They can only produce provisioning goods, not all the regulatory and supporting ecosystem services that complex agroecology landscapes provide through their aboveground and belowground biodiversity (Wilbois and Schmidt 2019). In addition to this, the materials and energy required to build and operate these agricultural factories raise serious concerns about their sustainability and viability on a large scale (Slameršak et al. 2022; Nieto et al. 2019; Krausmann et al. 2017). In any case, the worst agricultural final energy yield prospects seem to be trying to merge the two way-outs along the diagonal line in Fig. 4, where all vectors are shorter from the origin vertex according to the optimality analysis performed. Society must decide the way forward. The approach presented here provides empirical and methodological grounds to inform such decisions, by identifying those pathways that combine the agroecological benefits of energetic circularity with the agronomic benefits of energetic efficiency. These prospective considerations based on the optimality analysis of the possible relationships that exist between the three EROIs of our circular energy modeling of farming go beyond the agroecosystem energy transition view that we proposed earlier (Gingrich et al. 2018b) and coincide with the same two options to address the dilemma of maximizing yields or energy efficiency pointed out by Carl F. Jordan (2016).

### Limits of our circular multi-EROI model and possibilities for further research

Models are useful tools for only a limited number of tasks. When we propose and use new ones, it is always good to explicitly warn of their limits not only to avoid misuse, but to help new research go further. Our circular approach has abandoned a single-minded notion of energy efficiency of complex systems, using multiple EROIs instead of one. The black box of the functioning of agroecosystems has begun to be opened, highlighting the role of the internal reuse of biomass as a reinvestment of farmers in the living funds’ reproduction. In doing so, we have followed Georgescu-Roegen’s (1971) distinction between biophysical “funds” and “flows” and placed the sustainability focus on their relationship: how much is given to the agroecosystem living funds in relation to what is taken out from them. However, we recognize that we end up summarizing the long-term paths followed by the flow/flow values of three EROIs without delving too much into the flow/fund ones behind. And we also acknowledge that this means aggregating in the *EI*, *BR*, and *FP* values different types of energy flows of different power ranges, qualities, and reproductive functions for the different funds involved.

A combination of emergy and exergy analyses at farm and agroecosystem levels can tackle better than our material and energy flow accounting (MEFA) the latter energy aggregation problem, and the recent proposals made by Jean Hercher-Pasteur with other colleagues at the Institut Agro in Montpellier have started overcoming the previous linearity required to account for emergy transformities (Hercher-Pasteur 2020, Hercher-Pasteur et al. 2020a, b). The MuSIASEM proposal by Mario Giampietro and other ICTA colleagues (Giampietro et al. 2011, 2013) is the best-known approach to overcome at the same time the two main limitations of our MEFA approach. As put forward by Julien-François Gerber and Arnim Scheidel (2018), MuSIASEM is more integrative and comprehensive than MEFA from a flow-fund perspective, although MEFA can be more easily comparative and historical using EROIs. There are also further possibilities for our circular MEFA analysis of farm systems to advance, like the broader agroecological multi-EROI proposal of Gloria Guzmán and Manuel González de Molina (2015, 2017).

## Conclusion

Our first research question aimed to discover what happens when agricultural energy balances are calculated not only for specific crops, but for entire agroecosystems from past organic to current industrial farming. We mapped for the first time in a multi-EROI possibility surface the changing energy profiles of 82 North American and European agroecosystems throughout the long-term transition from traditional organic to full industrial agriculture. Through this energy mapping and statistical analysis, we conclude that the prevailing path has led them into an energy trap of low energy returns on external inputs with little or no increase in the returns on internal inputs or all inputs consumed.

Our second research question sought to unravel what role the disintegration between the cropland, livestock, and forestry components of agroecosystems has played in this energy trap (Fig 1). Statistical analysis has led us to conclude that in our case studies the sharp increases in external non-renewable inputs, with only minor or no reductions in internal biomass reuse, were driven primarily by the dietary transition and by forestry reduction or abandonment. Both have entailed deep structural changes in the composition of agroecosystems and the energy carriers that flow in and out of them. The functional disintegration among cropland, livestock, pastures, and forests has led to linear agroecosystem flows increasingly driven towards a very inefficient feed-to-meat energy bioconversion. Together with the declining significance of energy efficient forestry, these structural changes of agroecosystems also explain the poor energy performance of industrial agriculture in the Global North.

This article reveals for the first time the importance of a circular integration between the components of a farm system for the energy performance counted at the agroecosystem level. According to these analyses and results, a sustainable way out of the energy trap of industrial agriculture will be to manage agroecosystems so that farmers reinvest once more in the internal cycles of nature. These cycles integrate the living funds of agroecosystems in a more circular biophysical turnover capable to upgrade their energy efficiency, reduce GHG emissions, improve soil fertility and carbon sequestration, prevent water pollution, and keep the supporting and regulating ecosystem services that biodiversity provides (Dainese et al. 2019; Van der Ploeg et al. 2019; Migliorini and Wezel 2017). The agroecological multi-EROI energy analysis applied in this study is a contribution to this task.

### Supplementary Information

Below is the link to the electronic supplementary material.

Supplementary file1 (XLS 586 KB)

Supplementary file2 (DOCX 1980 KB)

## Data Availability

All data used in this analytical synthesis of the multi-EROI dataset assembled by the international SFS project from 2012 to 2022 in North American and European countries and regions can be found in the Table SM1 and the Excel file of the Supplementary Material. The detailed data for each flow and farm system component collected for these 82 energy balances are explained in the references of each case study and three methodological working papers (Aguilera et al. 2015; Cunfer et al. 2018; Díez-Sanjuán et al. 2018; Fraňková and Cattaneo 2018; Fullana et al. 2021; Galán et al 2016; Gingrich and Krausmann 2018; Gingrich et al. 2018a; Gómez 2017; Guzmán and González de Molina 2008, 2015; Guzmán et al. 2014; Guzmán et al. 2018; Marco et al. 2018; McFadyen and Watson 2018; Padró et al. 2017; Parcerisas and Dupras 2018; Soto et al. 2016; Tello 2015; Tello et al. 2016). All the EROIs used here have been recalculated from the disaggregated data of all energy flows listed in Table 1 provided by all co-authors for the 19 case studies from 5 countries. In some cases, this has meant some adjustments or improvements to the first data provided in previous publications.
