# Supplementary material for: Assessing the energy trap of industrial agriculture in North America and Europe: 82 balances from 1830 to 2012
Source: Agron Sustain Dev. 2023 Nov 8;43(6):75. doi: 10.1007/s13593-023-00925-5 (PMC10632262; doi:10.1007/s13593-023-00925-5)
Supplement: Supplementary file 2 — Supplementary file2 (DOCX 1980 KB) [file 13593_2023_925_MOESM2_ESM.docx]

Supplementary Material

SMa Case study locations, empirical multi-EROI values plotted in the energy map of FEROI-IFEROI-EFEROI variations, and variables used in the statistical analyses

Fig. SM1 Location of the case study sites.


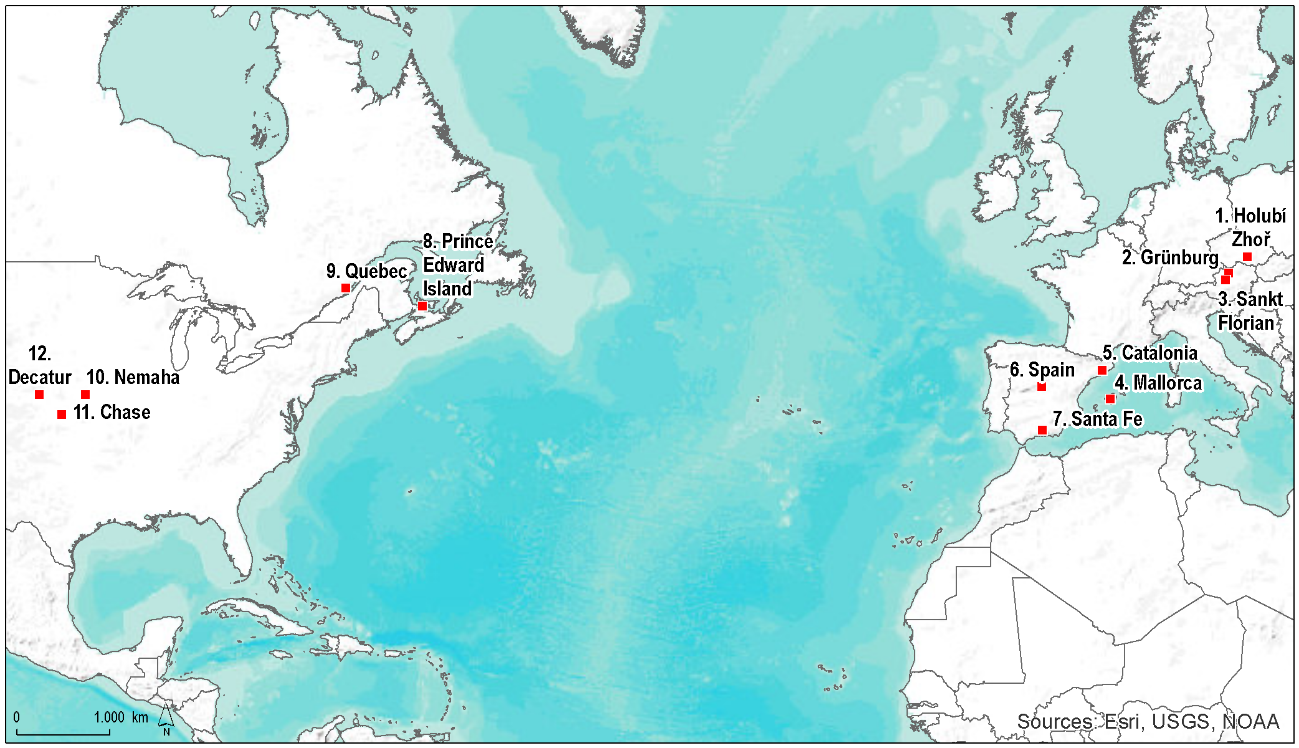


Source: Our own (see references in the availability of data and material of the article, and Table SM1 in this Supplementary Material).

Table SM1 The SFS multi-EROI dataset of agricultural systems in North America and Europe (1830-2012).

| **Coun try** | **County or District**  **(Province or State)** | **Municipality** | **Year** | ***FEROI*** | ***IFEROI*** | ***EFEROI*** | **FP/ha** | **L/ha** | **LV/ ha** | **W/ha** | **LV–FP** | **W–FP** | **WS** |
| --- | --- | --- | --- | --- | --- | --- | --- | --- | --- | --- | --- | --- | --- |
| US | Nemaha (Kansas) |  | 1880 | 0.23 | 0.29 | 1.28 | 5.01 | 0.04 | 0.48 | 0.00 | 0.10 | 0.00 | 0.05 |
| US | Nemaha (Kansas) |  | 1930 | 0.29 | 0.33 | 2.35 | 7.63 | 0.04 | 1.02 | 0.00 | 0.13 | 0.00 | 0.04 |
| US | Nemaha (Kansas) |  | 1954 | 0.46 | 0.56 | 2.59 | 13.12 | 0.03 | 0.56 | 0.00 | 0.04 | 0.00 | 0.05 |
| US | Nemaha (Kansas) |  | 1997 | 0.68 | 0.97 | 2.27 | 26.71 | 0.01 | 0.92 | 0.00 | 0.03 | 0.00 | 0.05 |
| US | Chase (Kansas) |  | 1880 | 0.20 | 0.24 | 1.41 | 2.06 | 0.02 | 0.19 | 0.00 | 0.09 | 0.00 | 0.02 |
| US | Chase (Kansas) |  | 1930 | 0.34 | 0.40 | 2.08 | 4.11 | 0.01 | 0.32 | 0.00 | 0.08 | 0.00 | 0.02 |
| US | Chase (Kansas) |  | 1954 | 0.12 | 0.15 | 0.68 | 1.61 | 0.02 | 0.24 | 0.00 | 0.15 | 0.00 | 0.01 |
| US | Chase (Kansas) |  | 1997 | 0.16 | 0.20 | 0.88 | 3.66 | 0.00 | 0.40 | 0.00 | 0.11 | 0.00 | 0.01 |
| US | Decatur (Kansas) |  | 1880 | 0.14 | 0.17 | 0.81 | 0.26 | 0.01 | 0.02 | 0.00 | 0.08 | 0.00 | 0.00 |
| US | Decatur (Kansas) |  | 1930 | 0.35 | 0.39 | 3.74 | 4.88 | 0.02 | 0.39 | 0.00 | 0.08 | 0.00 | 0.00 |
| US | Decatur (Kansas) |  | 1954 | 0.42 | 0.50 | 2.50 | 5.80 | 0.02 | 0.24 | 0.00 | 0.04 | 0.00 | 0.01 |
| US | Decatur (Kansas) |  | 1997 | 0.57 | 0.77 | 2.18 | 12.99 | 0.00 | 0.33 | 0.00 | 0.03 | 0.00 | 0.01 |
| CA | Prince Edward Island (PEI) |  | 1880 | 1.24 | 1.41 | 10.17 | 21.61 | 0.17 | 0.12 | 20.33 | 0.01 | 0.94 | 0.46 |
| CA | Prince Edward Island (PEI) |  | 1930 | 0.94 | 1.04 | 9.94 | 19.93 | 0.10 | 0.66 | 17.10 | 0.03 | 0.86 | 0.37 |
| CA | Prince Edward Island (PEI) |  | 1950 | 0.88 | 1.02 | 6.42 | 18.08 | 0.10 | 0.54 | 14.46 | 0.03 | 0.80 | 0.47 |
| CA | Prince Edward Island (PEI) |  | 1996 | 1.44 | 2.03 | 5.00 | 30.73 | 0.02 | 1.27 | 13.52 | 0.04 | 0.44 | 0.58 |
| CA | Prince County (PEI) |  | 1880 | 1.54 | 1.78 | 11.50 | 23.78 | 0.16 | 0.10 | 22.62 | 0.00 | 0.95 | 0.49 |
| CA | Prince County (PEI) |  | 1930 | 0.92 | 1.01 | 10.31 | 19.39 | 0.11 | 0.58 | 16.65 | 0.03 | 0.86 | 0.34 |
| CA | Prince County (PEI) |  | 1950 | 0.83 | 0.96 | 6.07 | 17.78 | 0.11 | 0.58 | 14.91 | 0.03 | 0.84 | 0.44 |
| CA | Prince County (PEI) |  | 1996 | 1.67 | 2.43 | 5.35 | 33.46 | 0.01 | 1.10 | 13.27 | 0.03 | 0.40 | 0.52 |
| CA | Kings County (PEI) |  | 1880 | 1.39 | 1.76 | 6.65 | 20.52 | 0.15 | 0.12 | 19.58 | 0.01 | 0.95 | 0.56 |
| CA | Kings County (PEI) |  | 1930 | 1.10 | 1.24 | 9.83 | 18.64 | 0.09 | 0.70 | 15.97 | 0.04 | 0.86 | 0.49 |
| CA | Kings County (PEI) |  | 1950 | 1.13 | 1.34 | 7.26 | 15.82 | 0.08 | 0.70 | 12.78 | 0.04 | 0.81 | 0.62 |
| CA | Kings County (PEI) |  | 1996 | 2.30 | 3.40 | 7.16 | 23.15 | 0.01 | 1.33 | 13.00 | 0.06 | 0.56 | 0.76 |
| CA | Queens County (PEI) |  | 1880 | 0.93 | 0.99 | 14.75 | 20.31 | 0.19 | 0.13 | 18.64 | 0.01 | 0.92 | 0.35 |
| CA | Queens County (PEI) |  | 1930 | 0.87 | 0.95 | 9.83 | 21.57 | 0.11 | 0.71 | 18.48 | 0.03 | 0.86 | 0.31 |
| CA | Queens County (PEI) |  | 1950 | 0.79 | 0.92 | 5.43 | 20.29 | 0.11 | 0.37 | 15.41 | 0.02 | 0.76 | 0.37 |
| CA | Queens County (PEI) |  | 1996 | 0.78 | 1.03 | 3.16 | 24.33 | 0.02 | 1.38 | 12.78 | 0.06 | 0.53 | 0.48 |
| CA | Province of Quebec (QC) |  | 1871 | 1.97 | 2.58 | 8.41 | 12.96 | 0.12 | 0.00 | 5.91 | 0.00 | 0.46 | 0.32 |
| CA | Province of Quebec (QC) |  | 1931 | 0.37 | 0.50 | 1.38 | 7.34 | 0.50 | 0.00 | 3.16 | 0.00 | 0.43 | 0.35 |
| CA | Province of Quebec (QC) |  | 1951 | 0.36 | 0.59 | 0.92 | 10.72 | 0.66 | 0.00 | 3.81 | 0.00 | 0.36 | 0.35 |
| CA | Province of Quebec (QC) |  | 1981 | 0.18 | 0.55 | 0.28 | 20.60 | 0.21 | 0.00 | 7.22 | 0.00 | 0.35 | 0.28 |
| CA | Province of Quebec (QC) |  | 2011 | 0.17 | 0.54 | 0.24 | 29.99 | 0.12 | 0.00 | 8.26 | 0.00 | 0.28 | 0.31 |
| AT | Linz-Land (Upper Austria) | Sankt Florian (Asten, Ansfelden) | 1830 | 0.51 | 0.53 | 17.25 | 18.06 | 0.99 | 0.00 | 0.00 | 0.00 | 0.00 | 0.17 |
| AT | Linz-Land (Upper Austria) | Sankt Florian (Asten, Ansfelden) | 1864 | 0.43 | 0.44 | 19.91 | 27.06 | 1.25 | 0.00 | 0.00 | 0.00 | 0.00 | 0.18 |
| AT | Linz-Land (Upper Austria) | Sankt Florian (Asten, Ansfelden) | 1950 | 0.34 | 0.44 | 1.46 | 19.83 | 2.39 | 0.00 | 0.00 | 0.00 | 0.00 | 0.18 |
| AT | Linz-Land (Upper Austria) | Sankt Florian (Asten, Ansfelden) | 1960 | 0.56 | 0.68 | 3.14 | 39.71 | 1.92 | 0.00 | 0.00 | 0.00 | 0.00 | 0.19 |
| AT | Linz-Land (Upper Austria) | Sankt Florian (Asten, Ansfelden) | 2000 | 1.26 | 2.54 | 2.51 | 109.05 | 1.38 | 0.00 | 0.00 | 0.00 | 0.00 | 0.18 |
| AT | Kirchdorf an der Krems (Upper Austria) | Grünburg (Steinbach an der Steyr, Waldneukirchen, Adlwang) | 1830 | 0.58 | 0.60 | 17.88 | 14.66 | 0.81 | 0.00 | 0.00 | 0.00 | 0.00 | 0.28 |
| AT | Kirchdorf an der Krems (Upper Austria) | Grünburg (Steinbach an der Steyr, Waldneukirchen, Adlwang) | 1864 | 0.64 | 0.66 | 25.43 | 21.28 | 0.81 | 0.00 | 0.00 | 0.00 | 0.00 | 0.27 |
| AT | Kirchdorf an der Krems (Upper Austria) | Grünburg (Steinbach an der Steyr, Waldneukirchen, Adlwang) | 1950 | 0.33 | 0.39 | 2.20 | 16.90 | 2.51 | 0.00 | 0.00 | 0.00 | 0.00 | 0.22 |
| AT | Kirchdorf an der Krems (Upper Austria) | Grünburg (Steinbach an der Steyr, Waldneukirchen, Adlwang) | 1960 | 0.28 | 0.33 | 1.81 | 20.32 | 2.10 | 0.00 | 0.00 | 0.00 | 0.00 | 0.24 |
| AT | Kirchdorf an der Krems (Upper Austria) | Grünburg (Steinbach an der Steyr, Waldneukirchen, Adlwang) | 2000 | 0.32 | 0.63 | 0.63 | 41.48 | 2.03 | 0.00 | 0.00 | 0.00 | 0.00 | 0.31 |
| AT | Austria |  | 1850 | 1.09 | 1.11 | 53.60 | 12.85 | 0.24 | 0.52 | 11.45 | 0.04 | 0.89 | 0.41 |
| AT | Austria |  | 1880 | 0.84 | 0.86 | 45.97 | 11.18 | 0.24 | 0.60 | 9.71 | 0.05 | 0.87 | 0.42 |
| AT | Austria |  | 1900 | 0.69 | 0.70 | 38.72 | 9.90 | 0.26 | 0.75 | 7.91 | 0.08 | 0.80 | 0.42 |
| AT | Austria |  | 1950 | 0.65 | 0.68 | 17.95 | 10.46 | 0.20 | 0.87 | 8.37 | 0.08 | 0.80 | 0.45 |
| AT | Austria |  | 1990 | 0.68 | 0.78 | 5.37 | 23.09 | 0.03 | 1.95 | 17.95 | 0.08 | 0.78 | 0.53 |
| AT | Austria |  | 2010 | 0.97 | 1.14 | 6.42 | 27.83 | 0.02 | 2.02 | 22.04 | 0.07 | 0.79 | 0.54 |
| CZ | Žďár nad Sázavou (Kraj Vysočina) | Holubí Zhoř | 1840 | 0.28 | 0.30 | 7.50 | 8.80 | 0.59 | 0.95 | 3.40 | 0.11 | 0.39 | 0.04 |
| CZ | Žďár nad Sázavou (Kraj Vysočina) | Holubí Zhoř | 2012 | 0.62 | 0.90 | 1.96 | 16.90 | 0.38 | 0.35 | 10.11 | 0.02 | 0.60 | 0.30 |
| ES | Spain |  | 1900 | 0.78 | 0.82 | 17.26 | 8.84 | 0.40 | 0.20 | 5.08 | 0.02 | 0.57 | 0.06 |
| ES | Spain |  | 1910 | 0.72 | 0.76 | 14.31 | 9.06 | 0.40 | 0.24 | 5.06 | 0.03 | 0.56 | 0.06 |
| ES | Spain |  | 1920 | 0.70 | 0.75 | 11.83 | 9.72 | 0.40 | 0.29 | 5.20 | 0.03 | 0.54 | 0.07 |
| ES | Spain |  | 1930 | 0.65 | 0.69 | 10.83 | 10.22 | 0.40 | 0.32 | 5.10 | 0.03 | 0.50 | 0.08 |
| ES | Spain |  | 1940 | 0.59 | 0.63 | 11.12 | 9.16 | 0.40 | 0.27 | 4.98 | 0.03 | 0.54 | 0.08 |
| ES | Spain |  | 1950 | 0.57 | 0.61 | 9.24 | 9.33 | 0.40 | 0.31 | 4.80 | 0.03 | 0.52 | 0.08 |
| ES | Spain |  | 1960 | 0.53 | 0.60 | 4.80 | 9.61 | 0.40 | 0.44 | 4.11 | 0.05 | 0.43 | 0.11 |
| ES | Spain |  | 1970 | 0.55 | 0.79 | 1.80 | 9.57 | 0.33 | 0.76 | 3.15 | 0.08 | 0.33 | 0.13 |
| ES | Spain |  | 1980 | 0.45 | 0.74 | 1.12 | 9.64 | 0.26 | 1.17 | 2.06 | 0.12 | 0.21 | 0.14 |
| ES | Spain |  | 1990 | 0.52 | 0.77 | 1.56 | 11.79 | 0.20 | 1.55 | 2.93 | 0.13 | 0.25 | 0.15 |
| ES | Spain |  | 2000 | 0.45 | 0.71 | 1.22 | 12.71 | 0.14 | 2.11 | 2.91 | 0.17 | 0.23 | 0.16 |
| ES | Spain |  | 2008 | 0.45 | 0.72 | 1.20 | 13.19 | 0.07 | 2.28 | 3.55 | 0.17 | 0.27 | 0.18 |
| ES | Baix Maresme (Barcelona, CAT) |  | 1850 | 0.70 | 1.19 | 1.73 | 27.96 | 0.51 | 0.88 | 13.18 | 0.03 | 0.47 | 0.30 |
| ES | Baix Maresme (Barcelona, CAT) |  | 1950 | 0.89 | 1.62 | 1.98 | 41.71 | 0.50 | 0.45 | 18.15 | 0.01 | 0.44 | 0.33 |
| ES | Baix Maresme (Barcelona, CAT) |  | 2010 | 0.65 | 3.17 | 0.81 | 27.00 | 1.34 | 4.16 | 1.13 | 0.15 | 0.04 | 0.47 |
| ES | Alt Maresme (Barcelona, CAT) |  | 1850 | 1.03 | 1.29 | 5.03 | 24.34 | 0.44 | 0.34 | 13.55 | 0.01 | 0.56 | 0.30 |
| ES | Alt Maresme (Barcelona, CAT) |  | 1950 | 0.99 | 1.63 | 2.55 | 33.28 | 0.37 | 0.48 | 17.49 | 0.01 | 0.53 | 0.34 |
| ES | Alt Maresme (Barcelona, CAT) |  | 2010 | 0.46 | 0.87 | 0.96 | 23.51 | 0.68 | 6.30 | 1.28 | 0.27 | 0.05 | 0.57 |
| ES | Vallès (Barcelona, CAT) | Caldes de Montbui, Castellar del Vallès., Polinyà, Sentmenat | 1860 | 1.06 | 1.12 | 22.22 | 21.88 | 0.29 | 0.22 | 18.20 | 0.01 | 0.83 | 0.35 |
| ES | Valles (Barcelona, CAT) | Sentmenat | 1920 | 0.98 | 1.03 | 19.70 | 31.86 | 0.49 | 0.34 | 24.67 | 0.01 | 0.77 | 0.36 |
| ES | Vallès (Barcelona, CAT) | Caldes de Montbui, Castellar del Vallès., Polinyà, Sentmenat | 1956 | 1.01 | 1.63 | 2.65 | 19.67 | 0.36 | 0.62 | 8.77 | 0.03 | 0.45 | 0.42 |
| ES | Vallès (Barcelona, CAT) | Caldes de Montbui, Castellar del Vallès., Polinyà, Sentmenat | 1999 | 0.22 | 2.20 | 0.25 | 33.50 | 0.34 | 25.61 | 2.87 | 0.76 | 0.09 | 0.57 |
| ES | Segarra (Lleida, CAT) | Les Oluges | 1860 | 0.69 | 0.71 | 19.14 | 13.00 | 0.13 | 0.16 | 11.13 | 0.01 | 0.86 | 0.44 |
| ES | Segarra (Lleida, CAT) | Les Oluges | 1959 | 1.15 | 1.40 | 6.57 | 23.14 | 0.19 | 0.12 | 2.16 | 0.01 | 0.09 | 0.11 |
| ES | Segarra (Lleida, CAT) | Les Oluges | 1999 | 0.49 | 4.23 | 0.55 | 77.74 | 0.26 | 16.78 | 0.09 | 0.22 | 0.00 | 0.16 |
| ES | Vega de Granada (AND) | Santa Fe | 1904 | 1.02 | 1.24 | 5.74 | 26.81 | 0.27 | 1.91 | 1.73 | 0.07 | 0.06 | 0.01 |
| ES | Vega de Granada (AND) | Santa Fe | 1934 | 0.76 | 0.89 | 5.14 | 27.84 | 0.24 | 2.34 | 8.16 | 0.08 | 0.29 | 0.06 |
| ES | Vega de Granada (AND) | Santa Fe | 1997 | 0.85 | 1.37 | 2.23 | 77.51 | 0.11 | 3.13 | 34.65 | 0.04 | 0.45 | 0.11 |
| ES | Llevant (Mallorca, Balearic Islands) | Manacor | 1860 | 0.51 | 0.55 | 7.09 | 5.35 | 0.27 | 0.19 | 2.71 | 0.04 | 0.51 | 0.40 |
| ES | Llevant (Mallorca, Balearic Islands) | Manacor | 1956 | 0.46 | 0.53 | 3.24 | 14.01 | 0.90 | 1.08 | 6.32 | 0.08 | 0.45 | 0.25 |
| ES | Llevant (Mallorca, Balearic Islands) | Manacor | 2012 | 0.47 | 1.34 | 0.71 | 19.84 | 0.62 | 1.10 | 2.92 | 2.92 | 0.06 | 0.15 |

Note: FP/ha: Final Produce in GJ per farmland hectare; L/ha: Human Labor in GJ per farmland hectare; LV/ha: Livestock Produce in GJ per farmland hectare; W/ha: Woodland Produce in GJ per farmland hectare; LV–FP: Share of Livestock Produce/Final Produce; W–FP: Share of Woodland Produce/Final Produce; WS: Share of Woodland area/farmland area. Sources: see the availability of data and material of the article and the Excel file of the Supplementary Material.

Fig. SM2 *FEROI* historical trends in the 82 agroecosystems of North America and Europe (1830-2012).

Source: Our own from the data shown in Table SM2. Black lines are interpolations between time points accounted.

Fig. SM3 *IFEROI* historical trends in the 82 agroecosystems of North America and Europe (1830-2012).

Source: Our own from the data shown in Table SM2. Black lines are interpolations between time points accounted.

Fig. SM4 *EFEROI* historical trends in the 82 agroecosystems of North America and Europe (1830-2012).

Source: Our own from the data shown in Table SM2. Black lines are interpolations between time points accounted.

SMb Further explanations on theory, hypotheses, and calculation methods

*A caveat on the economic fallacy of perfect input substitution*

A methodological novelty of this article is to present the three EROIs accounted for the 82 agricultural energy balances here analyzed plotted as coordinates within the surface of possible values that they can take according to the equation (4) that relates them (i.e., the final energy return of an agroecosystem is equal to the product of its external and internal energy returns divided by their sum). This multi-EROI analysis has three key differences with the presentation of the entire database in the usual time series for each EROI, as they are shown in the graphs included in the previous part of this Supplementary Material (Figs. SM2, SM3 and SM4). First, the three-dimensional possibility surface tells us what values ​​these *FEROI-IFEROI-EFEROI* can simultaneously take and not. Second, through the curvature of this three-dimensional surface an optimization analysis can be performed to show which combinations of *IFEROI-EFEROI* increases are the most efficient to attain an additional increase in *FEROI* at any point of that possibility surface (for the method used, and its demonstration by Vera Sacristán, see the annex of Tello et al. 2016). And thirdly, the optimal paths can be compared with the real ones. Thanks to that, we can map the actual transition paths in a way that informs on the role that the changing proportions of external inputs and internal biomass reuses per unit of the final product extracted from the agroecosystems play in each case. It also helps to figure out some possible opposite options in the roadmap to overcome the current energy trap of the fossil-based industrial agriculture, as explained in section 3.3 of the article.

This novel multi-EROI energy analysis of agroecosystems focuses on the respective role of internal biomass reuses and external matter-energy inputs as drivers of the socioecological transition from traditional organic to industrial farming. That is the strength and usefulness of the model. However, as in any other model, it also sets its own limits as briefly pointed out in section 3.3. of the article. We know, on theoretical and empirical grounds, that the reproduction of organic agroecosystems is based mainly on the internal circulation of renewable biomass flows while industrial agriculture is highly dependent on external inputs mainly coming from fossil fuels at present. However, we also know that the real possibilities of substituting one for the other are limited, and that when these substitutions take place, they also involve structural changes in the agroecosystem functioning with strong environmental, social, and economic impacts on other dimensions not included in the model. Although the model as such does not assume that the reuse of renewable biomass flows (*BR*) and External Inputs (*EI*) of fossil origin are perfect substitutes for each other, it is important remembering that they are not when using it.

Therefore, when interpreting the actual shifts experienced in the $\frac{EI}{BR}$ ratios of the total amount of energy flows managed by farmers we must keep in mind the qualitative changes they imply from an agroecological point of view (Guzmán and González de Molina 2015 and 2017). Replacing internal biomass reuses (*BR*) by external inputs (*EI*) modifies the structural pattern of the living funds that allow the agroecosystem reproduction and affects its sustainability (Padró et al. 2019, 2020).

This limitation of the multi-EROI model stems from the problematic but unavoidable assumption made in energy analysis that involves counting in Joules the energy content of different energy carriers that flow in, out and within agroecosystems as if they were equal—i.e., setting aside their diverse qualitative nature and different power ranges (Jordan 2016; Giampietro, Mayumi and Sorman 2011, 2013; Murphy et al. 2011). Most of them are biomass flows coming from photosynthesis, while others are abiotic chemical products or fossilized biomass coming from the lithosphere. Some are provided by self-reproducing living funds, e.g., human labor, animal work and soil biota. Others are industrial mechanical implements and agrochemicals, e.g., tractors, synthetic fertilizers, and pesticides (Altieri 2018; Guzmán and González de Molina 2017). The shifts experienced by the energy flows driven by farmers are registered in our ‘energy map’ without considering the qualitative and structural changes they entail for agroecosystems. Although this does not explicitly assume that *EI* and *BR* are perfect substitutes, it cannot consider that they are not the same at all from an agroecology point of view.

We must take this caveat in mind when using the possibility surface of equation (4) to map the changing energy profiles of agroecosystems before and after industrialization of agriculture (Figs. 3, 4, 5 and 6 of the article). Accounting these flows without caution, as if they simply meant a replacement of one energy carrier by another equivalent, would imply incurring in what in ecological economics is known as the fallacy of the perfect substitution between production factors (Martins 2016; Pelenc and Ballet 2015; Gómez-Baggethun et al. 2010; Dietz and Neumayer 2007; Daly 1990; Georgescu-Roegen 1976). Given that we are dealing with complex evolving agroecosystems that work in the interplay between society and nature, any sustainability assessment requires a multidimensional and multi-scalar nexus approach able to combine and integrate different dimensions (Giampietro, Mayumi and Sorman 2013).

As said in the article, and particularly in section 3.3, combining different models is the only way to unravel the structural changes between the components of the agroecosystem that interact through these dimensions and scales. This article contributes to this sustainability evaluation of the path that industrial agriculture has followed, but only from the energy dimension. All models are useful tools to carry out only some tasks but not others. In this circular multi-EROI approach, we use energy analysis to scan the main structural changes agroecosystems have experienced throughout the socioecological transition from past organic to current industrial agriculture.

*Additional information on the quantitative and statistical analyses*

- - 1. *Visualizing diverging cases within the FEROI-IFEROI-EFEROI possibility surface*

The two graphs shown in Fig. 5 of the article keep the proportionality among the values of the three axes to show the three-dimensional graphs or bidimensional energy maps of the *FEROI-EFEROI-IFEROI* possibility surface. However, in the sample used *EFEROI* values move from 0.24 to 53.6, whereas *IFEROI* only moves from 0.15 to 4.23 and *FEROI* from 0.12 to 2.30 (Table SM1 in this Supplementary Material). As a result, keeping the proportionality among axes makes it difficult to notice some cases whose differences appear too small in these figures, particularly among the cloud of points located in low *FEROI-EFEROI-IFEROI* scores near to their origin vertices. To help highlight these cases, in the graphs shown in Fig. SM5 the *EFEROI* axis has been expanded relative to the scale of the other two axes. This second two-dimensional energy map (where *FEROI* height values are shown by contour lines) includes interpolation lines between time points at the same locations.

**Fig. SM5** EROI results of the 82 agroecosystems studied plotted in the possibility surface of all FEROI-EFEROI-IFEROI values according to equation (4) in the article, this time shown by enlarging the EFEROI axis relative to the other two in order to better appreciate some particular diverging cases.


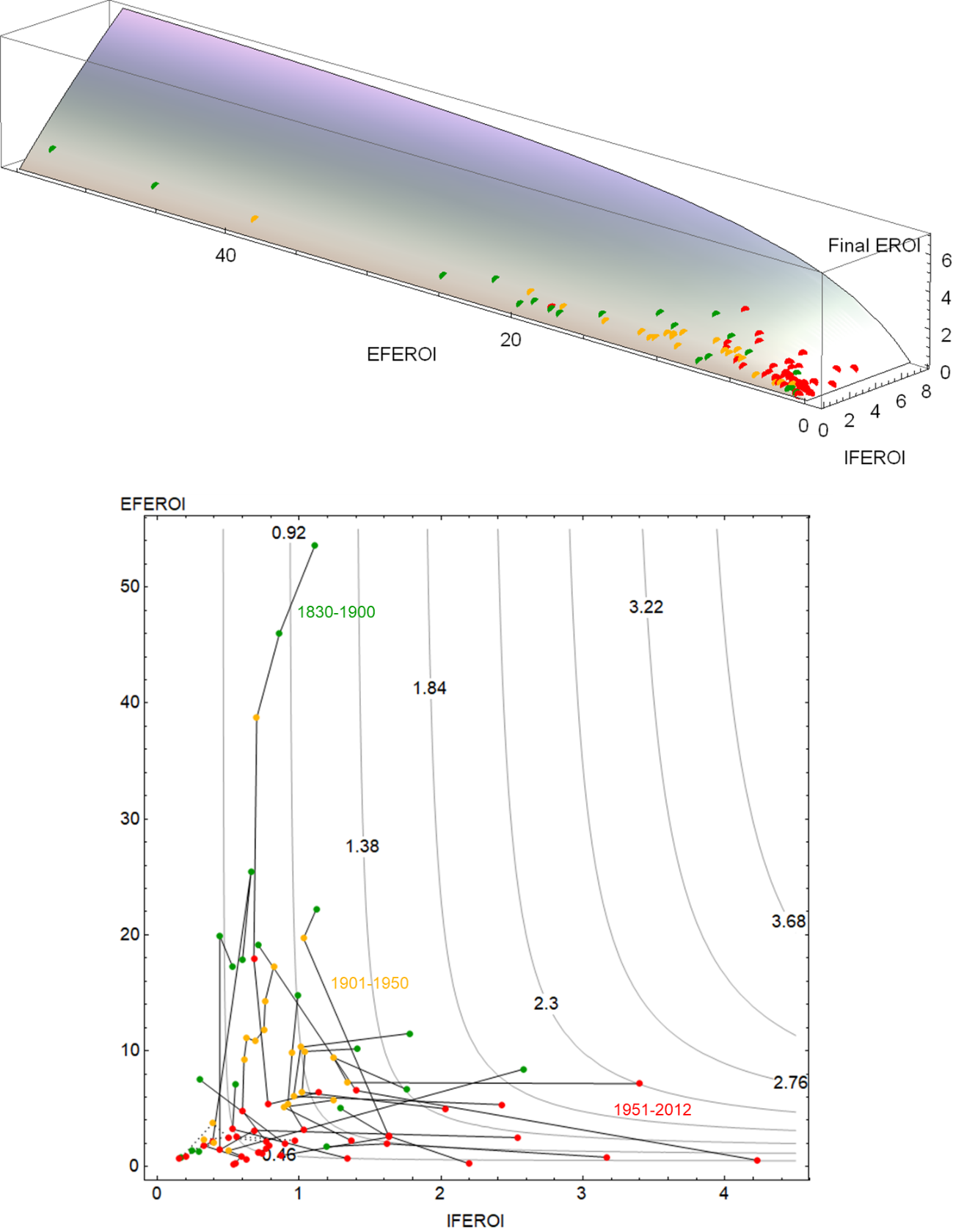


Straight lines on the lower bidimensional graph are interpolations between time points of the same case studies, and dashed lines help to highlight some diverging cases. Sources: Our own from the data shown in Table SM1.

- - 1. *More on the two statistical tests carried out with the SFS dataset*

The two statistical tests presented in the article are addressing different questions and complement each other. The first test looks for the main common drivers of *FEROI, IFEROI* and *EFEROI* variations in the entire dataset using linear mixed-effects models with nine independent variables, as fixed effects included when they comply with basic statistical assumptions and improve the AIC value by at least two units in relation to the null models. Each case study was introduced as a random effect nested within its country. To avoid biasing the data set, we limited to six the observations included in the sample of the multi-EROI annual historical series for Austria (Gingrich and Krausmann 2018). Data from the 2012 case of the Holubí Zhoř farm in the Czech Republic was removed in the *EFEROI* model because it behaved as an influential value (Fraňková and Cattaneo 2018). Temporal autocorrelation was avoided by introducing the year of the energy balance (*Y*) (Fig. SM6):

**Table SM2** Summary of the three mixed-effects models using as dependent variables log (FEROI), log (IFEROI), and log (EFEROI).

| mixed-effects log (*FEROI*) model:  $\log\left( FEROI \right)=2.71+0.01\cdot FP-2.65\cdot LV_{FP}+1.66\cdot WS-0.28\cdot L-0.002\cdot Y$  AIC model =121; AIC null model = 133 | | |
| --- | --- | --- |
| significant variables | Chi sq. | P (> Chi sq.) |
| *LV_FP* | 29 | < 0.001 |
| *WS* | 21 | < 0.001 |
| *FP* | 14 | < 0.001 |
| *L* | 7 | 0.01 |
| *Y* | 4 | 0.04 |
| mixed-effects log (*IFEROI*) model:  $\log(IFEROI)= -1.13+0.02\cdot FP+1.65\cdot WS$  AIC model = 98; AIC null model = 136 | | |
| significant variables | Chi sq. | P (> Chi sq.) |
| *FP* | 55 | < 0.001 |
| *WS* | 28 | < 0.001 |
| mixed-effects log (*EFEROI*) model:  $\log\left( EFEROI \right)=19.65-0.01\cdot Y+1.38\cdot W\_FP-2.82\cdot LV\_FP-0.50\cdot L$  AIC model = 199; AIC null model =264 | | |
| significant variables | Chi sq. | P (> Chi sq.) |
| *Y* | 37 | <0.001 |
| *W_FP* | 11 | <0.001 |
| *LP_FP* | 10 | 0.002 |
| *L* | 5 | 0.02 |

Sources: Our own from the data shown in Table SM1.

These first statistical mixed-effects models are leaving aside the specific geographic and historical characteristics of each agroecosystem that belongs to 19 different case studies located in different places (Fig. SM1), to focus on the main common drivers underlying the variation of *FEROI, IFEROI* and *EFEROI* values. ​Main common historical characteristics are addressed in the second t-test performed to check the statistical significance of the differences in the *FEROI, EFEROI* and *IFEROI* values ​​along the three periods of the socioecological transition from traditional organic to full industrial farming shown in different colors in our energy maps. As explained in the article, a t-test with a significance level of 0.05 is run between pairs of *FEROI*, *EFEROI* and *IFEROI* differences of the three periods (Table SM2).

**Table SM3** Main statistics of the FEROI-EFEROI-IFEROI values in the 82 energy balances of the three periods considered.

|  |  | Traditional Organic  1830-1900 | Intermediate  1901-1950 | Industrial  1951-2012 |
| --- | --- | --- | --- | --- |
| observations |  | 22 | 25 | 35 |
| min | *FEROI* | 0.14 | 0.29 | 0.12 |
|  | *EFEROI* | 0.81 | 1.38 | 0.24 |
|  | *IFEROI* | 0.17 | 0.33 | 0.15 |
| max | *FEROI* | 1.97 | 1.13 | 2.30 |
|  | *EFEROI* | 53.60 | 19.70 | 7.16 |
|  | *IFEROI* | 2.58 | 1.63 | 4.23 |
| mean | *FEROI* (*s.d.*) | 0.86 (*0.50*) A | 0.69 (*0.31*) A | 0.77 (*0.57*) A |
| (*standard* | *EFEROI* (*s.d.*) | 12.7 (*12.6*) A | 7.0 (*5.9*) A | 2.4 (*2.2*) BC |
| (*deviation*) | *IFEROI* (*s.d.*) | 1.01 (*0.64*) A | 0.85 (*0.44*) A | 1.64 (*1.13*) BC |

Sources: Our own from the data shown in Table SM1 and the references given the article. The key ABC after the mean category shows the results of the paired sampled t-tests, so that mean figures followed by the same letter in each of the three columns of results are not significantly different among periods according to the t-tests with a significance level of 0.05; n=15 for comparisons between the Traditional Organic and the Intermediate periods; n=15 for comparisons between the Intermediate and the Industrial periods; n=19 for comparisons between the Traditional Organic and the Industrial periods.

These statistical tests confirm that the steep *EFEROI* decrease has been the most relevant change of the long-term socioecological transition from solar-based traditional organic to current industrial agriculture.

**References**

Daly HE (1990) Sustainable Development: From Concept and Theory to Operational Principles. Popul Dev Rev16:25-43. <https://www.jstor.org/stable/2808061>

Dietz S, Neumayer E (2007) Weak and strong sustainability in the SEEA: Concepts and measurement. Ecol Econ 61:617-626. <https://doi.org/10.1016/j.ecolecon.2006.09.007>

Fraňková E, Cattaneo C (2018) Organic farming in the past and today: sociometabolic perspective on a Central European case study. Reg Environ Change 18(4):951-963. <https://doi.org/10.1007/s10113-016-1099-8>

Georgescu-Roegen N (1976) Energy and Economic Myths. Pergamon Press, New York.

Giampietro M, Mayumi K, Sorman AH (2011) The Metabolic Pattern of Societies. Where Economists Fall Short. Routledge, London.

Giampietro M, Mayumi K, Sorman AH (2013) Energy Analysis for Sustainable Future: Multi-Scale Integrated Analysis of Societal and Ecosystem Metabolism. Routledge, London.

Gingrich S, Krausmann F (2018) At the core of the socioecological transition: Agroecosystem energy fluxes in Austria 1830–2010. Sci The Total Environ 645(15):119-129. <https://doi.org/10.1016/j.scitotenv.2018.07.074>

Gómez-Baggethun E, de Groot R, Lomas PL, et al. (2010) The history of ecosystem services in economic theory and practice: From early notions to markets and payment schemes. *Ecol. Econ.* 69, 1209–1218. <https://doi.org/10.1016/j.ecolecon.2009.11.007>

Guzmán GI, González de Molina M (2015) Energy Efficiency in Agrarian Systems from an Agroecological Perspective. Agroecol Sust Food 39(8):924-952. <https://doi.org/10.1080/21683565.2015.1053587>

Guzmán GI, González de Molina M Eds (2017) Energy in Agroecosystems. A Tool for Assessing Sustainability. CRC Press, Boca Raton.

Jordan CF (2016). The Farm as a Thermodynamic System: Implications of the Maximum Power Principle. BioPhys Econ Res Qual 1:9. <https://doi.org/10.1007/s41247-016-0010-z>

Martins NO (2016) Ecosystems, strong sustainability and the classical circular economy. Ecol Econ 129:32-39. <https://doi.org/10.1016/j.ecolecon.2016.06.003>

Murphy DJ, Hall CAS, Dale M, et al. (2011) Order from Chaos: A Preliminary Protocol for Determining the EROI of Fuels. Sustainability 3:1888-1907. <https://doi.org/10.3390/su3101888>

Padró R, Marco I, Font C, et al. (2019) Beyond Chayanov: A sustainable agroecological farm reproductive analysis of peasant domestic units and rural communities (Sentmenat; Catalonia, 1860). Ecol Econ 160:227-239. <https://doi.org/10.1016/j.ecolecon.2019.02.009>

Padró R, Tello E, Marco I, et al. (2020) Modelling the Scaling Up of Sustainable Farming into Agroecology Territories: Potentials and Bottlenecks at the Landscape Level in a Mediterranean Case Study. J Clean Prod 275:124043. <https://doi.org/10.1016/j.jclepro.2020.124043>

Pelenc J, Ballet J (2015) Strong sustainability, critical natural capital and the capability approach. Ecol Econ 112:36-44. <https://doi.org/10.1016/j.ecolecon.2015.02.006>

Tello E, Galán E, Sacristán V, et al. (2016) Opening the black box of energy throughputs in agroecosystems: a decomposition analysis of final EROI into its internal and external returns (the Vallès County Catalonia, c.1860 and 1999). Ecol Econ 121:160-174. <https://doi.org/10.1016/j.ecolecon.2015.11.012>
